# The alternative splicing of intersectin 1 regulated by PTBP1 promotes human glioma progression

**DOI:** 10.1038/s41419-022-05238-1

**Published:** 2022-09-28

**Authors:** Chungen Lan, Huikun Zhang, Kezhen Wang, Xiaoli Liu, Yawen Zhao, Zhifang Guo, Ning Zhang, Yongxia Zhou, Manzhi Gao, Feng Gu, Yongjie Ma

**Affiliations:** 1 Department of Breast Cancer Pathology and Research Laboratory, Tianjin Medical University Cancer Institute and Hospital, Tianjin, China; 2 Department of Tumor Cell Biology, Tianjin Medical University Cancer Institute and Hospital, National Clinical Research Center for Cancer, Tianjin, China; 3 Tianjin’s Clinical Research Center for Cancer, Tianjin Medical University Cancer Institute and Hospital, Tianjin, China; 4 Key Laboratory of Cancer Prevention and Therapy, Tianjin, China; 5 Key Laboratory of Breast Cancer Prevention and Therapy, Tianjin Medical University, Ministry of Education, Tianjin, China

**Keywords:** CNS cancer, Oncogenes, Targeted therapies

## Abstract

Intersectin 1 (ITSN1) contains two isoforms: ITSN1-S and ITSN1-L, which are highly regulated by alternative splicing. Our previous results showed that the two isoforms of ITSN1 displayed opposite functions: ITSN1-S promoted glioma development, while ITSN1-L exerted an inhibitory role in glioma progression. In this study, our transcriptome analysis using a large glioma cohort indicated that the ratio of ITSN1-S/ITSN1-L was positively correlated with glioma grading and poor prognosis. We identified the RNA-binding protein polypyrimidine tract-binding protein 1 (PTBP1) as an *ITSN1* pre-mRNA interaction protein through RNA pull-down assay and RNA immunoprecipitation assay. Knockdown of PTBP1 decreased the ratio of ITSN1-S/ITSN1-L. Minigene reporter assay and mutation analyses further confirmed PTBP1 targeted polypyrimidine sequences on ITSN1 exon 30 (TTGCACTTCAGTATTTT) and promoted the inclusion of ITSN1 exon 30. Subsequently, silencing PTBP1 inhibited glioma cell proliferation, migration, and invasion by down-regulating the ratio of ITSN1-S/ITSN1-L. Taken together, our study provides a novel mechanism that PTBP1 modulates the alternative splicing of ITSN1 and promotes glioma proliferation and motility by up-regulating the ratio of ITSN1-S/ITSN1-L, thereby highlighting that PTBP1 may be an attractive therapeutic target for gliomas.

## Introduction

Glioma is the most common and fatal brain cancer in adults [1]. Despite aggressive therapies with surgical resection, radiotherapy, and chemotherapy, the treatment outcome is still not satisfactory due to the highly invasive nature of glioma [2, 3]. ITSN1 is a highly conserved scaffold protein during evolution with multiple domains [4]. Its two major isoforms regulated by alternative splicing of exon 30 [5], referred to as long isoform (ITSN1-L) and short isoform (ITSN1-S), displayed opposite roles in glioma progression according to our previous studies [6]. ITSN1-S promotes glioma cell proliferation, migration, and invasion by regulating several key proteins [7, 8], while ITSN1-L decreases the aggressive phenotype of glioma cells [6]. Correspondingly, we discovered by database analyses that the two isoforms were differentially expressed in gliomas and non-neoplastic brain tissues: ITSN1-L was highly enriched in non-neoplastic brain tissues, whereas ITSN1-S took the majority in gliomas. Therefore, upregulation of ITSN1-L expression and down-regulation of ITSN1-S expression simultaneously at an alternative splicing level probably is a better strategy in glioma treatment.

Alternative splicing is a post‐transcriptional modification that regulates transcriptome and proteome diversity [9, 10], resulting in protein isoforms with different or even opposite functions [11, 12]. Growing evidence has shown that alternative splicing is closely associated with tumorigenesis and progression [13, 14]. For instance, alternative splicing of the pyruvate kinase M gene produces the M2 isoform and promotes aerobic glycolysis and tumor growth [15]. These studies highlight the importance of understanding alternative splicing patterns in tumor progression.

Splicing factors involve a large variety of RNA-binding proteins (RBPs), such as heterogeneous nuclear ribonucleoproteins (hnRNPs) and serine/arginine-rich (SR) proteins [16]. Here we found PTBP1 (also known as hnRNP I) was capable of binding the pre-mRNA of *ITSN1* and silencing PTBP1 remarkably decreased the ratio of ITSN1-S/ITSN1-L. PTBP1, a member of hnRNPs, is an RBP that is repressed during normal neurogenesis to allow the differentiation of progenitor cells into mature neurons [17], but is highly expressed in brain tumors [18]. In this study, we identified the potential binding site of PTBP1 to ITSN1 exon 30 RNA and further confirmed that PTBP1 functionally interacted with the poly-pyrimidine sequences (TTGCACTTCAGTATTTT) to activate the inclusion of ITSN1 exon 30. Moreover, we demonstrated that the expression of PTBP1 was positively correlated with glioma grading and poor prognosis, and PTBP1 promoted glioma cell proliferation, migration, and invasion by increasing the ratio of ITSN1-S/ITSN1-L. Together with previous studies, our work illustrated that splicing control of the ratio of ITSN1-S/ITSN1-L is an important aspect in glioma progression and suggested the usage of PTBP1 as a promising therapeutic target for gliomas.

## Materials and methods

### Patients’ clinical information

118 glioma tissue samples were randomly selected. These patients were 74 men and 44 women who underwent surgery between 1999 and 2013. The mean age of the patients at the time of diagnosis was 46.9 years, ranging from 9 to 80 years. The use of human samples was approved by the Ethics Committee of Tianjin Medical University Cancer Institute and Hospital. The informed consent forms from all subjects were obtained. Based on the post-surgery pathology report, 4 (3.4%) samples were categorized as grade I, 41 (34.7%) samples were categorized as grade II, 25 (21.2%) samples were categorized as grade III, and 48 (40.7%) samples were categorized as grade IV. None of the patients had received radiotherapy or chemotherapy prior to surgery. Follow-up information was available for 118 patients, who were followed up until October of 2013. Histopathology review and diagnosis confirmation were performed by three independent pathologists in a blinded manner.

### Immunohistochemistry staining

Immunohistochemistry for ITSN1-S and Ki67 was performed using standard techniques by Streptavidin-Peroxidase method. Two neuropathologists evaluated their staining. Sections for ITSN1-S were classified as follows: low (0–30% of stained cells) and high (31–100% of stained cells). Immunostaining of Ki67 (Zymed, California, USA) was scored according to the percentage of tumor cells with positive nucleus staining. ITSN1-S antibodies were used as previously described [7].

### Cell culture

LN229, U87MG, 140, and HEK-293T cells were cultured in Dulbecco’s modified Eagle’s medium (DMEM) supplemented with 10% fetal bovine serum (FBS) in a 5% CO_2_ incubator at 37 °C. Cells were tested and authenticated in Beijing Microread Genetics Co., Ltd. by short tandem repeat profiling. All cell lines tested negative for mycoplasma contamination.

### Plasmid construction and transfection

For construction of shRNA expression plasmids, specific shRNA and scrambled sequences (Supplementary Table S1) were synthesized and cloned into pLKO.1 or pLVX vector, respectively. PCDH-mGFP-ITSN1-S and pCDH-mGFP-ITSN1-L plasmids were constructed previously [6]. An open reading frame clone of Homo sapiens full-length PTBP1 was amplified via polymerase chain reaction (PCR) with related primers, and then inserted into pCDH-CMV-MCS-EF1-Puro lentiviral vector. For in vitro transcription experiments, the sequence consisting of the full length of ITSN1 exon 30 as well as 300 nucleotides of intron 29 and 30 adjacent to exon 30 was amplified via PCR, and subcloned into pCDH vector. The resulting plasmid was named as pCDH-ITSN1-E30. For construction of ITSN1 wild-type minigene vector, an ITSN1 exon 29–30 with 300 bps intron 30 downstream of exon 30 DNA fragment and an ITSN1 exon 31 with 300 bps intron 30 upstream of exon 31 DNA fragment were PCR-amplified individually, and subcloned into pCDH vector by Trelief^TM^ SoSoo Cloning Kit Ver.2 (Beijing TsingKe Biotech Co., Ltd, China). The resulting plasmid was named as pCDH-ITSN1-minigene. Related primers were listed in Supplementary Table S2. The LN229 cell line with *ITSN1* gene knocked out was constructed by our research team previously [6]. Lentivirus production and infection were performed as previously described [7].

### Construction of mutant plasmid

For construction of pCDH-ITSN1-E30 mutant plasmid and pCDH-ITSN1-minigene mutant plasmid, primers ITSN1-exon30-mut-F and ITSN1-exon30-mut-R were synthesized. PCR amplification was carried out by using pCDH-ITSN1-E30 plasmid or pCDH-ITSN1-minigene plasmid as a template, respectively. Subsequently, the amplified product was digested with Dpn I at 37 °C for 1 h. Then the reaction product was transformed into DH5α competent cells. Positive clones were selected, and the plasmid was extracted after verification by sequencing.

### Western blot analysis

Briefly, the cellular lysates were prepared in 1 x sodium dodecyl sulfate (SDS) lysis buffer and then were resolved by SDS–polyacrylamide gel electrophoresis (PAGE) and transferred onto nitrocellulose membranes (Millipore, Billerica, MA, USA). The membranes were incubated overnight at 4 °C with the appropriate primary antibody: anti-ITSN1, anti-β-actin (sc-47778, Santa Cruz Technology), anti-PTBP1 (sc-56701, Santa Cruz Technology or 32-4800, Invitrogen), anti-hnRNP K (sc-28380, Santa Cruz Technology), anti-hnRNP A2/B1 (sc-374053, Santa Cruz Technology), anti-GFP (KM-8009, SunGene Biotech), anti-MMP-9 (A0289, ABclonal), and N-cadherin (sc-59987, Santa Cruz Technology). The membranes were then treated with secondary antibodies (Li-Cor Biosciences, Lincoln, NE, USA). Infrared signals were examined by using the Odyssey imaging system (Li-Cor Biosciences, Lincoln, NE, USA).

### In vitro transcription of RNAs

Firstly, the pCDH-ITSN1-E30 plasmid was linearized by Xbal and NotI and then the linearized product was used as substrate in the in vitro transcription reaction. In vitro transcription was performed using the HiScribe^TM^ T7 High Yield RNA Synthesis Kit (New England Biolabs) following the manufacturer’s directions.

### RNA pull-down assay

Pull-down assay was carried out with Pierce Magnetic RNA pull-down Kit (Thermo Fisher Scientific, USA) following manufacturer’s protocol. Samples were UV-crosslinked (254 nm) with a Stratalinker ultraviolet cross-linker for 10 min. The precipitated products were analyzed by liquid chromatography tandem mass spectrometry (LC-MS) or western blot. Streptavidin magnetic beads alone were served as negative controls.

### RNA immunoprecipitation assay

RNA immunoprecipitation assay was performed using the EZ-Magna RIP kit (Millipore), according to the manufacturer’s instructions, with slight modifications. Ultraviolet cross-linking was performed for 10 min. Immunoprecipitation was done with anti-PTBP1 antibody (32-4800, Invitrogen). Mouse IgG (Millipore, CS200621) was used as control. RNA was extracted from the immunoprecipitate and cDNA was synthesized with random primers.

### Silver staining and mass spectrometry

The eluted proteins from RNA pull-down assay were resolved by SDS-PAGE and silver stained with the Fast Silver Stain Kit (P0017S, Beyotime, China). Gel strips of ITSN1-E30 RNA and negative control were cut and subjected to LC-MS sequencing provided by the Beijing Genomics Institute, China.

### A minigene reporter for measurement of ITSN1 exon 30 splicing

For transfection of ITSN1 minigenes, LN229 cells were grown to approximately 50%~60% confluency in 3.5 cm dish and transfected with either 2 μg of the ITSN1 wild-type minigene or ITSN1 mutant minigene plasmid using X-tremeGENE HP DNA Transfection Reagent from Roche (Mannheim, Germany) according to the manufacturer’s protocol following silencing PTBP1 or not. Forty-eight hours after transfection, cells were collected to measure the skipping of ITSN1 exon 30.

### RNA extraction, RT–PCR, and RT–qPCR

Total RNA was isolated from cells using Trizol reagent (Invitrogen, USA) according to the manufacturer’s instructions. cDNA was synthesized by the RTase M-MLV (Takara, Shiga-ken, Japan) as described in the manufacturer’s protocol. PCR was performed using specific primers. Quantitation of ITSN1 transcripts was done by RT–qPCR using SYBR Green PCR Master Mix (TaKaRa, Shiga-ken, Japan). The sequences of the primers are shown in Supplementary Table S3.

### EdU (5-Ethynyl-2′-deoxyuridine) assay

Tumor cells were seeded into 24-well plates at a density of 30,000 cells per well. EdU staining was conducted using the BeyoClick^TM^ EdU Cell Proliferation Kit (C00788L, Beyotime, China). The stained cells were observed and photographed under an inverted microscope. The fluorescence intensity was measured by ImageJ software. All the immunofluorescence and microscopy experiments were performed blinded.

### Migration and invasion assays

Transwell insert assays were performed to assess the invasive/migratory capacities of the cells. Transwell chambers with polycarbonate membranes (8-μm pore size), were either uncoated (migration assay) or coated (invasion assay) with Matrigel matrix (#356234, Corning, NY, USA) in serum-free DMEM. 5 × 10^4^ cells (migration assay) or 1 × 10^5^ cells (invasion assay) were suspended in 200 μL serum-free medium, and were seeded into the upper chambers. Medium containing 2% FBS (500 μL) was added to the lower chamber as a chemotactic factor. Following a 12 h (migration assay) or 24 h (invasion assay) incubation period in a 37 °C incubator, the non-migrating/invading cells on the upper surface of the filter were carefully removed with a cotton swab. The cells on the lower surface of the membrane were fixed and stained with Giemsa solution and photographed under a microscope (Olympus, Tokyo, Japan) at ×200 magnification.

### Tumor xenograft experiments

Four-week-old male BALB/c nude mice were purchased from Vital River Laboratory (Beijing, China). The animal experiment procedures were approved by the Ethics Committee of Tianjin Medical University Cancer Institute and Hospital. All studies were conducted in accordance with the principles and procedures outlined in the NIH Guide for the Care and Use of Laboratory Animals. The mice were randomized into different groups and used for subcutaneous transplantation. 1.8 × 10^6^ cells were injected subcutaneously into the left and right franks of each mouse (7–8 mice/group). The volume of tumors was calculated every week. Tumor volumes were calculated with the equation *V* = *L* × *W*^2^ × 0.5, where *L* is length and *W* is width. Mice were euthanized 7 or 8 weeks post-implantation, and tumors were removed and fixed with 4% paraformaldehyde. All tumor specimens were embedded in paraffin and stained with anti-Ki67 antibody. Hematoxylene and eosin (HE) staining was performed on the transplanted tumors to observe the invasion of the transplanted tumors. The invasion of transplanted tumors was analyzed as previously described [19–21].

### Bioinformatics analyses

To examine the expression of ITSN1-S, ITSN1-L, and the ratio of ITSN1-S/ITSN1-L in glioma, the glioma dataset from TCGA (http://gdac.broadinstitute.org) and GSE56517 dataset from Gene Expression Omnibus (GEO) database were downloaded and used for analyses. The Gene Expression Profiling Interactive Analysis (GEPIA) website (http://gepia.cancer-pku.cn/) was applied to analyze the 25 overlapped RBPs in LGG and GBM. GSE4290 dataset was used for analysis of the expression of the 25 overlapped RBPs in different grades of gliomas. PTBP1 binding sites were predicted using PTBP1 cross-linking IP sequencing (CLIP-seq) data and visualized by Integrative Genomics Viewer. Gene expression data and corresponding clinical data from 693 glioma patients were downloaded from Chinese Glioma Genome Atlas (CGGA) (http://www.cgga.org.cn/) and used for analyses.

### Statistical analyses

The GraphPad Prism version 8.0 and the SPSS version 22.0 were used for statistical analysis. Correlations between two variables were evaluated by Spearman’s rank-correlation test or Pearson correlation analysis. Overall survival (OS) rates were assessed using the Kaplan–Meier method, and the log-rank test was applied to compute *P* values. ANOVA was applied to compute *P* values in differential expression analysis in GEPIA and it was performed for group comparisons. At least three independent experiments were performed for all in vitro cellular functional experiments. All data were presented as mean ± SD, and Student’s *t*-test was used to compare the statistical significance between groups. A two-sided *P* < 0.05 was considered statistically significant.

## Results

### ITSN1-S and ITSN1-L displayed opposite roles in glioma progression and the ratio of ITSN1-S/ITSN1-L was positively correlated with glioma grading and poor prognosis

**Fig. 1:**
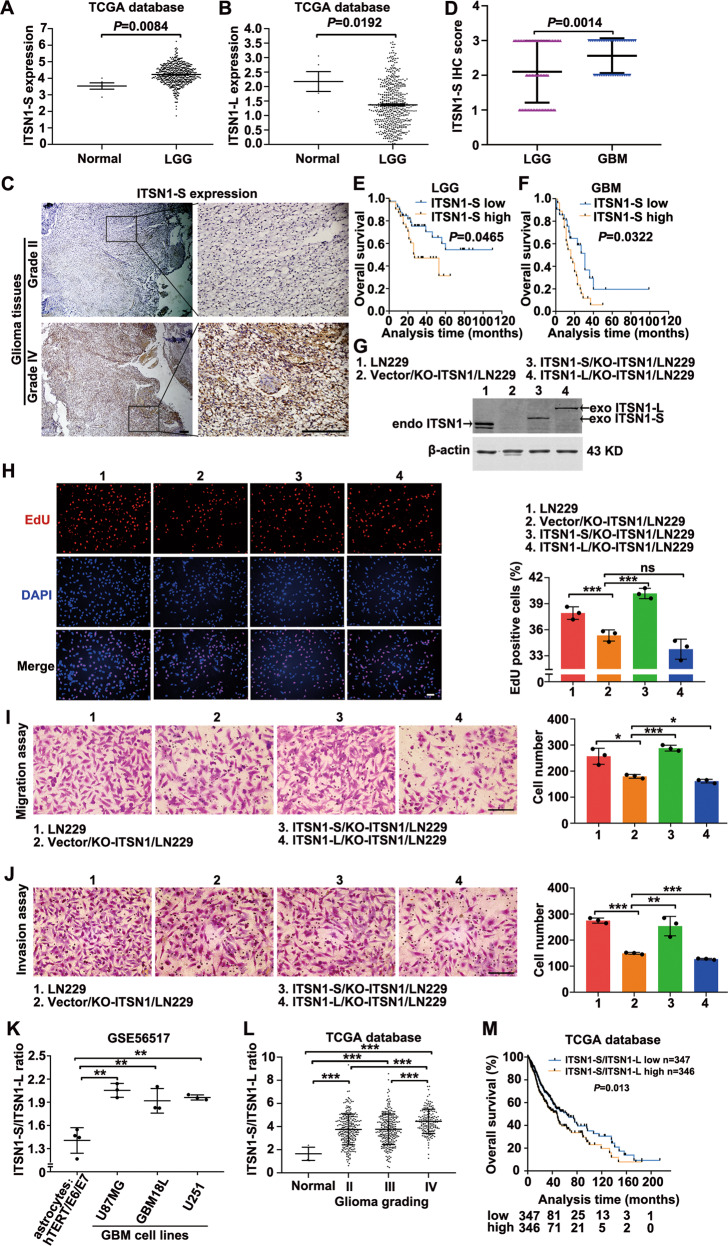
ITSN1-S and ITSN1-L displayed opposite roles in glioma progression and the ratio of ITSN1-S/ITSN1-L was positively correlated with glioma grading and poor prognosis. **A**, **B** Scatter plot comparing ITSN1-S (**A**) or ITSN1-L (**B**) mRNA levels in transcripts per million (TPM) between normal (*n* = 5) and LGG tissues (*n* = 528) using the data from TCGA dataset. The y axis represented the transcript expression levels measured by log_2_(TPM + 1). **C** Representative images of immunohistochemical staining of ITSN1-S in human glioma tissues with different histological grade. Scale bars, 200 μm. **D** The immunohistochemistry score of ITSN1-S in LGG (*n* = 70) and GBM (*n* = 48) based on our patient samples (*P* value was calculated by two-tailed Student’s *t* test). **E** Kaplan–Meier analyses of the OS of patients with LGG stratified into high- (*n* = 31) and low-expression (*n* = 39) groups by ITSN1-S expression. The *P* value of the log-rank (Mantel-Cox) test was presented. **F** Kaplan–Meier analyses of the OS of patients with GBM stratified into high- (*n* = 27) and low-expression (*n* = 21) groups by ITSN1-S expression. The *P* value of the log-rank (Mantel-Cox) test was presented. **G** ITSN1 knockout cells stably infected with lentiviruses of vector, mGFP-ITSN1-S and mGFP-ITSN1-L, respectively, were analyzed by western blot. **H** EdU assay was used to detect proliferation ability of the indicated cells. Scale bars, 100 μm. **I** Migration assay results. Cells migrating through transwell inserts were stained, photographed, and quantified. Scale bars, 200 μm. **J** Invasion assay results. Cells invading through matrigel matrix-coated transwell inserts were stained, photographed, and quantified, scale bars, 200 μm. Values were expressed as mean ± SD from three independent experiments in (**H**–**J**) (two-tailed Student’s *t* test, *P < 0.05, **P < 0.01, ***P < 0.001, ns, no significance). **K** Scatter plot comparing the mRNA expression ratio of ITSN1-S/ITSN1-L in immortalized human astrocytes and GBM cell lines (two-tailed Student’s *t* test, **P < 0.01). **L** The mRNA expression ratio of ITSN1-S/ITSN1-L in normal and different grades of glioma tissues using the data from TCGA datasets (two-tailed Student’s *t* test, ***P < 0.001). **M** Kaplan–Meier analyses of the OS of all glioma patients stratified into high- (*n* = 346) and low-ratio (*n* = 347) groups by ITSN1-S/ITSN1-L expression ratio.

Analysis of TCGA database identified the mRNA expression of two isoforms of ITSN1 in glioma. Figure 1A showed that ITSN1-S mRNA level in low-grade glioma (LGG) tissues was higher than that in normal tissues. In contrast, the ITSN1-L mRNA level in LGG tissues was lower than that in normal tissues (Fig. 1B). In order to validate the role of ITSN1-S expression in glioma development, the immunohistochemical analysis was conducted. We discovered that the expression of ITSN1-S was positively associated with tumor grade (*r*_s_ = 0.189, *P* = 0.041, Supplementary Table S4). Figure 1C showed representative images of different expression patterns of ITSN1-S in grade II and IV glioma tissues. Moreover, the immunohistochemistry score of ITSN1-S in patients with glioblastoma (GBM) was significantly higher than those with LGG (Fig. 1D). Notably, patients with a high level of ITSN1-S had significantly shorter overall survival than those with a low level of ITSN1-S in LGG (Fig. 1E) and GBM (Fig. 1F).

To further discover the roles of ITSN1-S and ITSN1-L played in glioma progression, lentivirus with full length of ITSN1-S and ITSN1-L were infected into LN229 cells with *ITSN1* gene knockout. Expression of ITSN1-S and ITSN1-L were examined by western blot (Fig. 1G). EdU assay showed that ITSN1-S overexpression promoted cell proliferation, which further confirmed our previous findings [7], while ITSN1-L overexpression had little effect on cell growth (Fig. 1H). Meanwhile, ITSN1-S overexpression promoted glioma cell migratory and invasive capacity, whereas overexpression of ITSN1-L inhibited the motility of glioma cell (Fig. 1I, J).

Further, we investigated whether the ratio of ITSN1-S/ITSN1-L was correlated with glioma progression. We found that the mRNA expression ratio of ITSN1-S/ITSN1-L in GBM cell lines was higher than that in immortalized astrocytes (Fig. 1K), and that the expression ratio of ITSN1-S/ITSN1-L increased with glioma grading by analysis of TCGA database (Fig. 1L). Moreover, the patients with a higher ITSN1-S/ITSN1-L expression ratio had a worse prognosis (Fig. 1M).

### Three candidate RBPs were capable of binding exon 30 and adjacent intronic areas of ITSN1 RNA

**Fig. 2:**
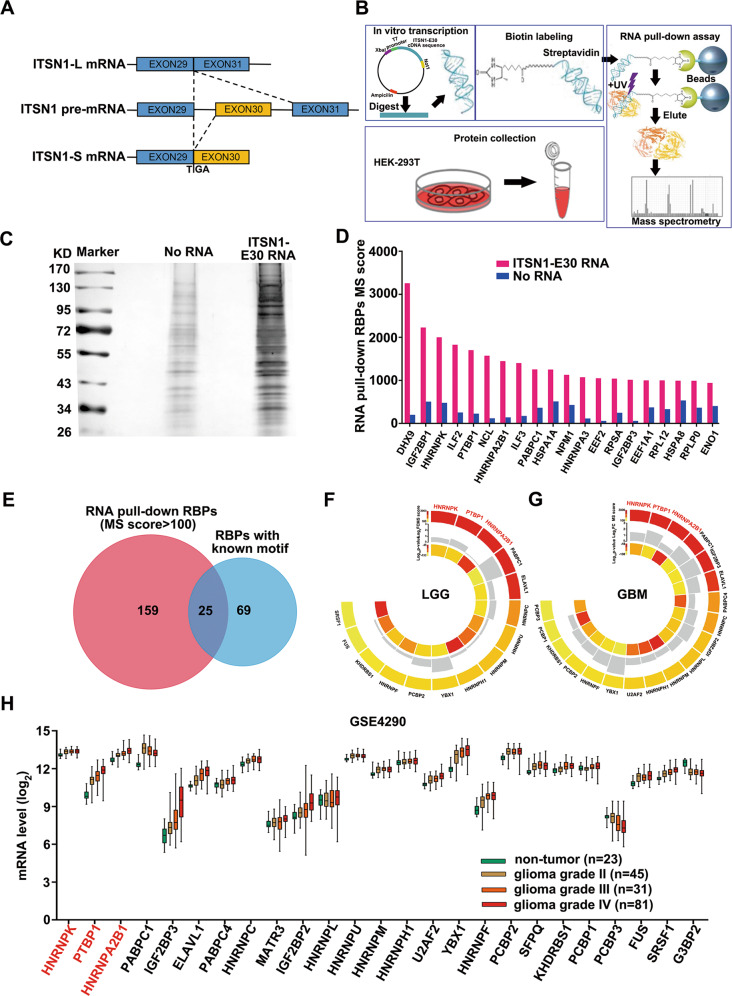
Three candidate RBPs were capable of binding exon 30 and adjacent intronic areas of ITSN1 RNA. **A** Schematic representation of the two isoforms of human ITSN1 resulting from an alternative splicing. **B** Schematic diagram of RNA pull-down assay and proteomics approaches. **C** In vitro RNA pull-down assay. Proteins were subjected to SDS-PAGE and silver stained after pull-down. **D** The top 20 RBPs ranked by MS score of RNA group. **E** The Venn diagram showed 25 overlapped RBPs between 184 RNA pull-downed RBPs (MS score >100) and 94 RBPs with known binding motifs. **F**, **G** Circos plots showed the differential expression analysis of the 25 overlapped RBPs in LGG (**F**) or GBM samples (**G**) and their paired normal samples from GEPIA (cut-off: |log_2_FC| > 1, *q*-value < 0.01). **H** A box plot showed the expression profile of the overlapped RBPs across non-neoplastic samples and grade II-IV glioma samples.

As is generally known, different isoforms of ITSN1 are highly regulated by alternative splicing [5]. Thus, we focused on identifying the specific splicing factor that regulates the alternative splicing of ITSN1. The splicing of exon 30 is critical for the formation of the two isoforms of ITSN1 (Fig. 2A). In addition, most of the cis-regulatory features that are predictive of tissue-dependent splicing patterns are located within ~300 nucleotides of the splice sites [22], the RNA sequence consisting of the full length of ITSN1 exon 30 as well as 300 nucleotides of intron 29 and 30 adjacent to exon 30 (named as ITSN1-E30) was used to perform the RNA pull-down assay. Work-flow of the whole experiment was shown in Fig. 2B. The RNA-protein complexes after pull-down were subjected to SDS-PAGE followed by silver staining (Fig. 2C). Gel strips of ITSN1-E30 RNA and negative control were cut and subjected to LC-MS.

The top 20 RBPs ranked by the MS score of the RNA group were shown in Fig. 2D, proteins without RNA-binding functions according to Uniprot (https://www.uniprot.org/) were neglected. Afterward, the 184 RBPs with an MS score over 100 from the RNA group were taken intersection with 94 RBPs from RBP map (http://rbpmap.technion.ac.il/) with already known binding motifs (Fig. 2E). 25 overlapping RBPs were identified (details shown in Table 1). Due to ITSN1-S/ITSN1-L ratio was altered in gliomas and non-neoplastic brain tissues, as well as different grades of glioma, we set out to investigate which of them manifested the same tendencies. Firstly, differential expression analysis of the 25 overlapping RBPs between LGG or GBM and paired normal samples was conducted in GEPIA (Fig. 2F, G, details shown in Supplementary Tables S5, S6). Moreover, to corroborate the relevance of these 25 selected RBPs with glioma grading, we evaluated their expression levels in non-neoplastic samples and grade II, III, and IV glioma samples from GSE4290 dataset (Fig. 2H). Analyses above determined 3 RBPs (PTBP1, hnRNP A2/B1, and hnRNP K) as candidates for ITSN1 splicing factors.

**Table 1:** The binding motifs of the 25 overlapped RBPs and their numbers of matches with ITSN1-E30 RNA sequence.

Gene name	Motifs				References	MS score		Matching sequence	Number of matches
						ITSN1-E30 RNA	No RNA		
HNRNPK		CCAWMCC			PMID: 19258514	2002	480	CCAWMCC	1
PTBP1	CUCUCU	UCUU	UUCUCU		PMID: 19296853	1703	227	UUCUCU/UCUU	1/11
HNRNPA2B1				UAGG	PMID: 23846655, PMID: 22574288	1448	140	UAGG	1
PABPC1					PMID: 23846655	1254	364	AAAAA	5
IGF2BP3					PMID: 23846655	1014	58	—	—
ELAVL1					PMID: 23846655	879	—	UUGGUUU	1
PABPC4					PMID: 23846655	577	221	—	—
HNRNPC					PMID: 23846655	545	187	UUUUU	18
MATR3					PMID: 23846655	459	30	—	—
IGF2BP2					PMID: 23846655	457	—	AAAACA	1
HNRNPL					PMID: 23846655	452	—	CACACA	1
HNRNPU					PMID: 22574288	442	89	UGUAGAG/UGUAUAG	1/2
HNRNPM					PMID: 22574288	425	66	—	—
HNRNPH1					PMID: 22574288	400	157	GAGGAG	1
U2AF2					PMID: 23846655	389	37	UUUUUC/UUUUCC	3/4
YBX1		CAACCACAA			PMID: 11222770, PMID: 19296853	326	67	AACACCA	1
HNRNPF			GGGUG	UUAGG	PMID: 19296853, PMID: 22574288	305	—	UUAGG	1
PCBP2					PMID: 10075886	301	35	CCUUCCC	1
SFPQ					PMID: 23846655	255	17	—	—
KHDRBS1					PMID: 23846655	210	—	AUAAAA	1
PCBP1					PMID: 10075886	202	46	—	—
PCBP3					PMID: 23846655	173	—	UUUCCC	2
FUS					PMID: 23846655	113	—	—	—
SRSF1		CRSMSGW	UGRWGVH		PMID: 19561594, PMID: 19296853	104	—	—	—
G3BP2					PMID: 23846655	103	—	—	—

### Knockdown of PTBP1 downregulated the ratio of ITSN1-S/ITSN1-L

**Fig. 3:**
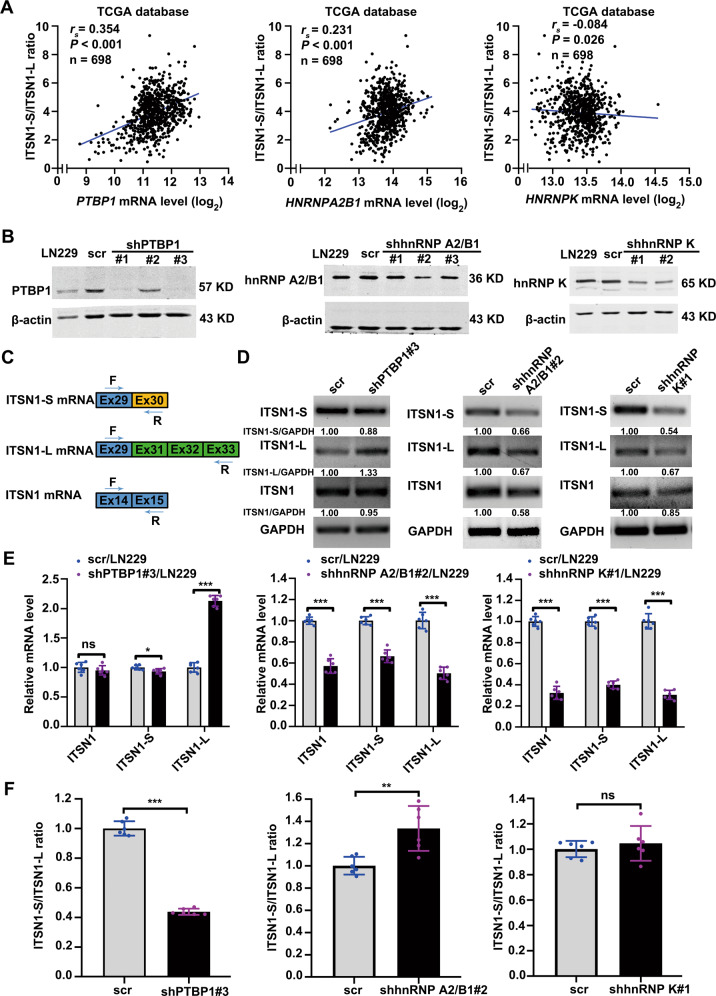
Knock-down of PTBP1 downregulated the ratio of ITSN1-S/ITSN1-L. **A** Scatter plot indicating the correlation of mRNA level of PTBP1, hnRNP A2/B1, and hnRNP K with ITSN1-S/ITSN1-L expression ratio using glioma RNA-Seq data from TCGA database. **B** Establishment and verification of stable cell lines underexpressing the 3 RBPs above. β-actin was used as an internal control. **C** Graphical representation of PCR primers for specific detection of ITSN1 and ITSN1-S, ITSN1-L isoforms. **D** RT–PCR detection of knockdown of the 3 RBPs above in LN229 cells on the expression levels of ITSN1, ITSN1-S, and ITSN1-L. **E**, **F** RT–qPCR analysis showing changes in the ITSN1-S, ITSN1-L, and total ITSN1 mRNA level (**E**) as well as the ratio of ITSN1-S/ITSN1-L (**F**) upon silencing of the 3 RBPs above in LN229 cells. Values were expressed as mean ± SD from six independent experiments in (**E**) and (**F**) (two-tailed Student’s *t* test, *P < 0.05, **P < 0.01, ***P < 0.001, ns, no significance).

Correlations between the mRNA levels of the three candidates and the ratio of ITSN1-S/ITSN1-L were examined in 698 glioma patient samples from TCGA database (Fig. 3A). PTBP1 and hnRNP A2/B1 were positively correlated with the ratio of ITSN1-S/ITSN1-L, respectively, while hnRNP K was negatively correlated with the ratio of ITSN1-S/ITSN1-L. And the correlation coefficient between PTBP1 and ITSN1-S/ITSN1-L ratio was the highest (*r*_s_ = 0.354). To determine the role of the three candidates in the exon 30 splicing of *ITSN1* pre-mRNA, PTBP1, hnRNP A2/B1, and hnRNP K were individually knocked down in LN229 cell line (Fig. 3B). Then, we designed specific primers for the detection of ITSN1 and ITSN1-S, ITSN1-L isoforms (Fig. 3C). The quantitative results of RT–PCR demonstrated that compared with control cells, the expression of ITSN1-L was significantly increased, while the expression level of ITSN1-S was slightly decreased in cells where PTBP1 was silenced. And PTBP1 knockdown didn’t affect the total expression level of ITSN1. Whereas silencing both hnRNP A2/B1 and hnRNP K could effectively down-regulate the expression of ITSN1-S, ITSN1-L, and total ITSN1 (Fig. 3D). Then RT–qPCR assays were performed to further verify these results. As shown in Fig. 3E, F, hnRNP A2/B1 knockdown simultaneously downregulated ITSN1-S and ITSN1-L but up-regulated the ratio of ITSN1-S/ITSN1-L. While knock-down of hnRNP K downregulated ITSN1-S and ITSN1-L simultaneously but showed no significant influence on the ratio of ITSN1-S/ITSN1-L. Only knock-down of PTBP1 lowered the ratio of ITSN1-S/ITSN1-L significantly by remarkably increasing ITSN1-L as well as slightly decreasing ITSN1-S, consistent with the results shown in U87MG and 140 (a primary glioma cell line) cell lines (Supplementary Fig. S1) as well as in LN229 cell line (Supplementary Fig. S2). These results indicated that PTBP1 may be a splicing factor of ITSN1.

### PTBP1 bound exon 30 and adjacent intronic areas of ITSN1 RNA and modulated splicing of ITSN1

**Fig. 4:**
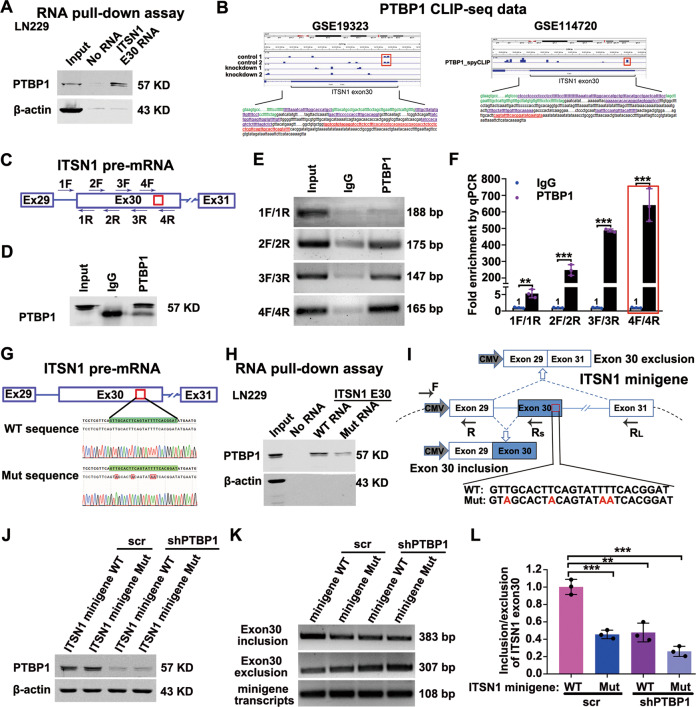
PTBP1 bound exon 30 and adjacent intronic areas of ITSN1 RNA and modulated splicing of ITSN1. **A** In vitro RNA pull-down assay in LN229 cell line. Proteins were subjected to western blot analysis with anti-PTBP1 antibody after pull-down. **B** Analysis of PTBP1 CLIP-seq data from GSE19323 and GSE114720, the blue lane shows PTBP1 CLIP peaks for exon 30 and adjacent area of introns of ITSN1. **C** The primer position used to detect the binding segment of PTBP1 and *ITSN1* pre-mRNA in the RIP experiment. **D** PTBP1 protein precipitation upon RIP with anti-PTBP1. **E** ITSN1 amplification from the anti-PTBP1 coprecipitated RNA fraction. **F** RIP with anti-PTBP1 followed by RT–qPCR quantification, values were expressed as mean ± SD from three independent experiments (two-tailed Student’s *t* test, **P < 0.01, ***P < 0.001). **G** The potential binding sequences of PTBP1 within ITSN1 E30 RNA used in the RNA pulldown assay and its position in the *ITSN1* pre-mRNA, the red font represented the mutated base. **H** RNA pulldown assay was used to detect the binding of PTBP1 to the biotinylated ITSN1 E30 WT RNA and ITSN1 mutant RNA. **I** Schematic representation of the ITSN1 minigene and the primer sets for PCR detection of ITSN1 exon 30-included and -excluded products. The primers for the internal reference of exogenous ITSN1-minigene were CMV-F and E29-R. The mutated base sequence of the ITSN1-minigene was marked with red font. **J** Western blot analysis for the inhibition of PTBP1 in transient cells transfected with the wild-type and mutant ITSN1-minigene plasmids. **K** Representative pictures of agarose PCR gels depicting the inversion in the splicing pattern of ITSN1 regulated by PTBP1 after transfected with the wild-type and mutant ITSN1-minigene plasmids. **L** RT–qPCR analysis showing changes in the ratio of ITSN1-S/ITSN1-L upon silencing PTBP1 in LN229 cells expressing ITSN1 minigene or ITSN1 mutant minigene. Values were expressed as mean ± SD from three independent experiments (two-tailed Student’s *t* test, ***P < 0.001).

In the following, the binding of PTBP1 and ITSN1-E30 RNA needed to be further confirmed. The results in Fig. 4A confirmed the existence of PTBP1 within ITSN1-E30 RNA pull-downed samples in LN229 cell line. In order to determine the exact binding section of PTBP1 and ITSN1-E30 RNA, we analyzed PTBP1 CLIP-seq data (Fig. 4B). Interestingly, there was one overlapping binding motif marked with a red frame between 2 control groups in GSE19323 and PTBP1_spyCLIP group in GSE114720. The overlapping binding sequence was displayed in red front with underscore. It suggested that the overlapping binding motif was the specific binding position of PTBP1 and ITSN1-E30 RNA. We then designed primers in several possible binding sequences including the overlapping binding sequence and performed RNA immunoprecipitation (RIP) assay to detect PTBP1 binding (Fig. 4C). IP of PTBP1 efficiently coprecipitated ITSN1 E30 RNA, confirming a direct interaction (Fig. 4D, E). As expected, RT–PCR from IgG RIP didn’t yield amplification of *ITSN1* pre-mRNA. Conversely, ITSN1 transcript was detected in the cDNA library of the PTBP1 RIP, and these results revealed that PTBP1 bound to the sequence between 4F and 4R in exon 30 of *ITSN1* pre-mRNA with higher affinity (Fig. 4E). Meanwhile, RT–qPCR results showed that ITSN1 RNA was significantly enriched in the PTBP1 RIP compared with IgG, and notably, the binding of PTBP1 to the sequences between 4F and 4R was more favorable than that to other binding sequences (Fig. 4F). In summary, these results confirmed that PTBP1 could bind ITSN1 exon 30 RNA.

Previous studies have reported that the consensus sequences for PTBP1 binding contain poly-pyrimidines. We found the potential binding sequence TTGCACTTCAGTATTTT (named exon 30-WT) for PTBP1 via pyrimidines sequence analysis. To further illustrate the interaction between PTBP1 and the potential binding site, the exon 30-WT sequence was mutated to TAGCACTACAGTATAAT (exon 30-Mut) (Fig. 4G). The results of RNA-pull down assay showed that PTBP1 could strongly bind with exon 30-WT RNA, but not exon 30-Mut RNA (Fig. 4H). To investigate the function of PTBP1 in alternating the splicing pattern of *ITSN1* pre-mRNA, we constructed an ITSN1 minigene plasmid and an ITSN1 minigene mutant plasmid that mutated the PTBP1 binding site. Then ITSN1 minigene or minigene mutant plasmid was transfected into LN229 cells with the inhibition of PTBP1 or not. RT–PCR with CMV-specific primers was performed to analyze the ITSN1 minigene-specific splicing products (Fig. 4I). The knock-down efficiency of PTBP1 was verified (Fig. 4J). Static levels of the inclusion and exclusion of ITSN1 exon 30 were examined by PCR-amplification followed by agarose gel electrophoresis (Fig. 4K). RT–PCR results indicated that both silencing PTBP1 and transfection of minigene mutant plasmid decreased the inclusion of ITSN1 exon 30 in ITSN1 minigene reporter systerm. Moreover, combination of silencing PTBP1 and transfection of minigene mutant plasmid further decreased the inclusion of ITSN1 exon 30. In parallel, RT–qPCR showed the same results (Fig. 4L). These results further confirmed that PTBP1 modulates splicing of ITSN1 by promoting the inclusion of ITSN1 exon 30.

### PTBP1 promoted glioma progression

**Fig. 5:**
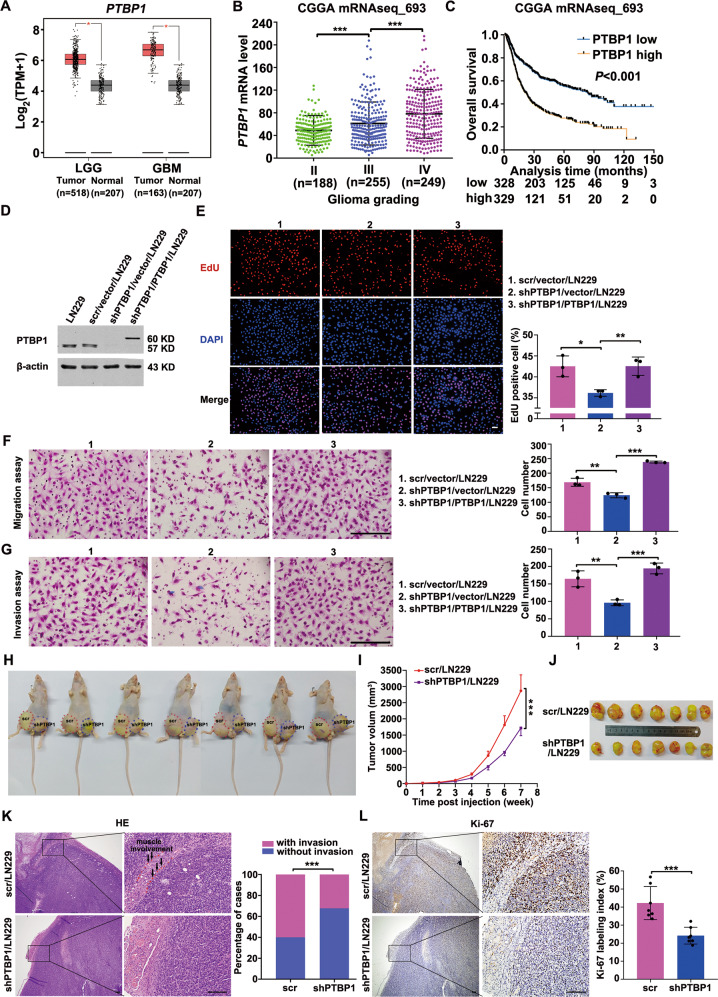
PTBP1 promoted glioma progression. **A** PTBP1 expression was evaluated between LGG or GBM and normal samples by GEPIA. **B** Scatter plot comparing *PTBP1* mRNA levels between different grades of glioma using the data from CGGA. **C** Curves of Kaplan–Meier analysis. OS was analyzed according to the PTBP1 expression (cut-off at 50% of the entire group). Low PTBP1 expression was related to long OS (*P* < 0.001). **D** Western blot analysis of PTBP1 knockdown and rescue in the extracts of cells as indicated. Loading control: β-actin. **E** Images of EdU staining (left, scale bars, 100 μm) and the comparison of EdU-positive rates between the control and silencing PTBP1 cells (right). **F** Migration assay of the indicated cells. Cells migrating through transwell inserts were stained, photographed, and quantified. Scale bars, 200 μm. **G** Invasion assay results. Cells invading through matrigel matrix-coated transwell inserts were stained, photographed, and quantified. Scale bars, 200 μm. Values were expressed as mean ± SD from three independent experiments in (**E**–**G**) (two-tailed Student’s *t* test, *P < 0.05, **P < 0.01, ***P < 0.001). **H** Nude mice injected with control or silencing PTBP1 LN229 cells. The images shown represent results for 7 mice. Red and blue dotted lines indicated the location and size of the tumors. **I** Growth curve of nude mice tumors within 7 weeks, values were expressed as mean ± SD (two-way ANOVA, ***P < 0.001). **J** The representative images show the xenograft tumors isolated from nude mice at the 7th week of tumor inoculation. **K** Representative images of hematoxylin-eosin staining (left), scale bars, 200 μm. The frequency of xenograft invasion in mice was analyzed quantitatively (right), *P* value was calculated using the chi-square test. ***P < 0.001. **L** Immunohistochemical images of tumor sections stained for Ki67 from control or PTBP1 knock down mice (left), quantitation of the percentage of Ki67-positive cells in tumor sections (right). Values are mean ± SD, two-tailed Student’s *t* test, ***P < 0.001. Scale bars, 200 μm.

To investigate whether PTBP1 plays a similar role in the malignant progression of glioma as the ratio of ITSN1-S/ITSN1-L, the expression analysis of PTBP1 in glioma tissues was conducted in GEPIA. The expression of PTBP1 was higher in both LGG and GBM compared with normal samples (Fig. 5A). Furthermore, in 693 glioma patient samples from CGGA, *PTBP1* mRNA level was positively correlated with glioma grading (Fig. 5B). And the patients with higher PTBP1 expression had a worse prognosis (Fig. 5C).

Then, we knocked down PTBP1 in LN229 cell line using shRNA targeting the 3’UTR of *PTBP1* mRNA and further we infected the PTBP1 knockdown cell line with a lentivirus co-expressing PTBP1 to restore PTBP1 expression (Fig. 5D). The results showed that PTBP1 knockdown inhibited proliferation, migration and invasion of LN229 cells. In addition, all functional defects could be rescued by PTBP1 restoration, confirming that these phenotypes were specifically due to PTBP1 depletion (Fig. 5E–G). Consistent with the results above, PTBP1 knockdown severely impaired the proliferation, migration, and invasion abilities, while PTBP1 restoration significantly rescued the above defects in U87MG cells (Supplementary Fig. S3). Next, the function of PTBP1 was also confirmed in vivo. Stable cell lines with PTBP1 silencing or not were injected subcutaneously into nude mice and formed tumors (Fig. 5H). Compared with control cells, tumors formed with PTBP1-silenced cells grew significantly slower (Fig. 5I). Then xenograft tumors were dissected 7 weeks post-inoculation. On gross inspection, the tumors formed by PTBP1 knockdown LN229 cells were markedly smaller compared with those formed by control cells (Fig. 5J). And total RNA was extracted from xenograft tumor tissues to detect the effect of silencing PTBP1 on the expression of ITSN1-S and ITSN1-L. RT–qPCR results showed that silencing PTBP1 lowered the ratio of ITSN1-S/ITSN1-L significantly by increasing ITSN1-L as well as decreasing ITSN1-S (Supplementary Fig. S4), which was consistent with the in vitro results. HE staining was applied in xenograft paraffin specimens and frequency of xenograft invasion was counted and determined. Notably, the expression level of PTBP1 was significantly positively correlated with tumor cell invasion (Fig. 5K). Epithelial–mesenchymal transition is a vital process in malignant tumor invasion [23]. The expression level of mesenchymal marker N-cadherin and epithelial marker E-cadherin were determined. Western blot analysis showed that silencing PTBP1 significantly downregulated the expression of N-cadherin (Supplementary Fig. S5). However, the expression of E-cadherin was too low to be detected in glioma xenograft tissues. Besides, processes of tumor cell invasion into the stromal tissues are closely related to the interactions between tumor cells and the extracellular matrix [24]. Matrix metalloproteases MMP-2 and MMP-9 were reported to be participated in the processes [25, 26]. As shown in Supplementary Fig. S5, MMP-9 expression was significantly inhibited after silencing PTBP1, while MMP-2 expression remained almost unchanged (data not shown). Moreover, the expression of Ki-67 was significantly decreased in shPTBP1 group compared with control group (Fig. 5L). These results demonstrated that PTBP1 could promote glioma progression in vivo and in vitro.

### PTBP1 promoted glioma progression by regulating alternative splicing of ITSN1

**Fig. 6:**
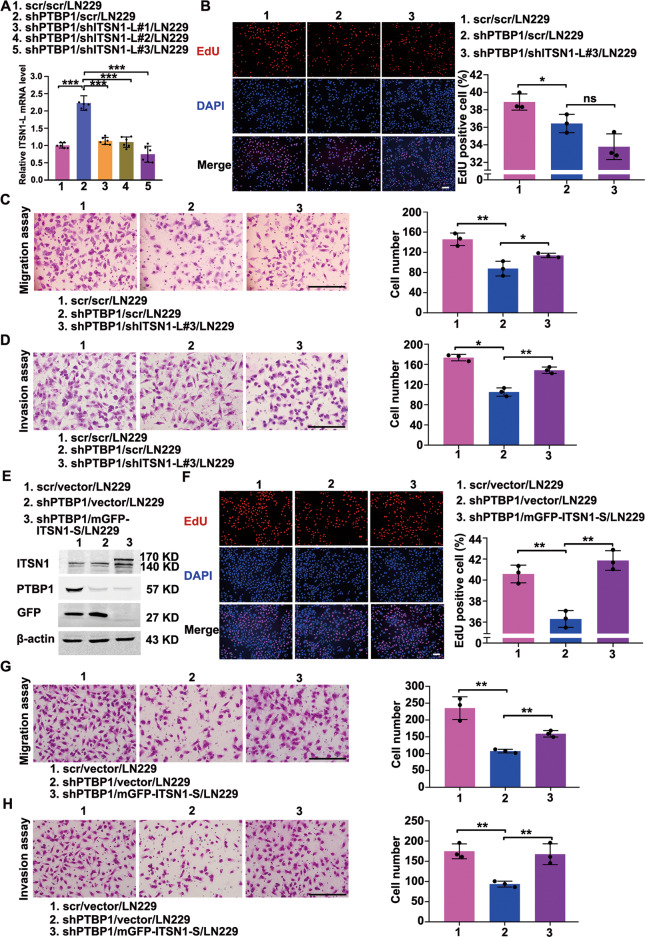
PTBP1 promoted proliferation, migration, and invasion of glioma cells by regulating alternative splicing of ITSN1 in vitro. **A** Establishment and verification of stable cell lines silencing PTBP1 followed by knocking down of ITNS1-L, Values were expressed as mean ± SD from six independent experiments (two-tailed Student’s *t* test, ***P < 0.001). **B** Images of EdU staining (left, scale bars, 100 μm) and the comparison of EdU-positive rates among indicated cells (right). **C** Migration assay of the indicated cells. Cells migrating through transwell inserts were stained, photographed, and quantified. Scale bars, 200 μm. **D** Invasion assay results. Cells invading through matrigel matrix-coated transwell inserts were stained, photographed, and quantified. Scale bars, 200 μm. **E** Western blot analysis of stable cell lines silencing PTBP1 followed by overexpression of ITSN1-S. **F** Images of EdU staining (left, scale bars, 100 μm) and the comparison of EdU-positive rates among indicated cells (right). **G** Migration assay of the indicated cells. Cells migrating through transwell inserts were stained, photographed, and quantified. Scale bars, 200 μm. **H** Invasion assay of the indicated cells. Cells invading through matrigel matrix-coated transwell inserts were stained, photographed, and quantified. Scale bars, 200 μm. Values were expressed as mean ± SD from three independent experiments in (**B**–**D**) and (**F**–**H**) (two-tailed Student’s *t* test, *P < 0.05, **P < 0.01, ns, no significance).

Given the regulation of ITSN1 splicing by PTBP1, we then investigated whether PTBP1 induces glioma progression by modulating the ratio of ITSN1-S/ITSN1-L in the form of increasing ITSN1-S while decreasing ITSN1-L. ITSN1-L was knocked down on top of PTBP1 silencing, and cells were named as shPTBP1/shITSN1-L/LN229 (Fig. 6A). EdU assay showed that ITSN1-L knockdown didn’t manifest significant impact on the proliferation of glioma cells based on PTBP1 silencing (Fig. 6B), while silencing of ITSN1-L could efficiently reverse the adverse effects of PTBP1 knockdown on glioma cells migration and invasion (Fig. 6C, D). Besides, we knocked down PTBP1 expression followed by up-regulating ITSN1-S with an mGFP tag, and cells were named as shPTBP1/mGFP-ITSN1-S/LN229 (Fig. 6E). We found that restoration of ITSN1-S could efficiently rescue the inhibitory effect of shPTBP1 on the proliferation, migratory and invasive abilities of glioma cells (Fig. 6F–H). Besides, consistent results were obtained in U87MG cells (Supplementary Fig. S6).

**Fig. 7:**
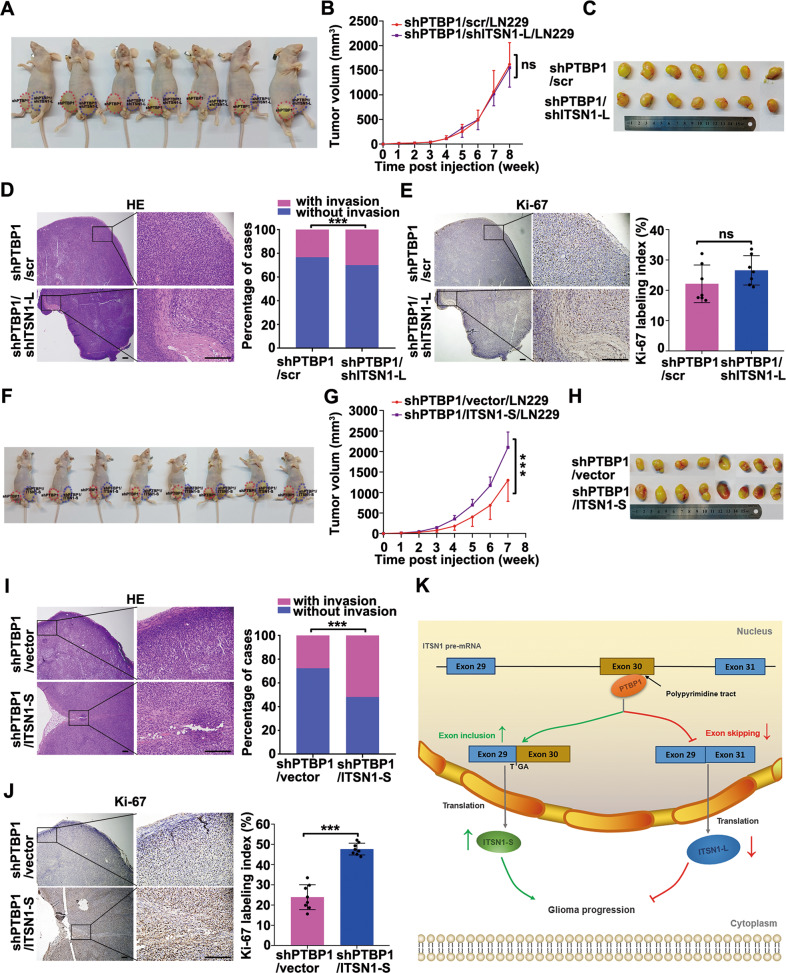
PTBP1 promoted proliferation, migration, and invasion of glioma cells by regulating alternative splicing of ITSN1 in vivo. **A** Nude mice injected with silencing PTBP1 or silencing PTBP1 combined with inhibition of ITSN1-L LN229 cells. The images shown represent results for 7 mice. Red and blue dotted lines indicated the location and size of the tumors. **B** Tumor growth curve in indicated nude mice. values were expressed as mean ± SD (two-way ANOVA, ns, no significance). **C** The representative images show xenograft tumor tissues isolated from the nude mice. **D** Representative images of hematoxylin-eosin staining (left, scale bars, 200 μm), the frequency of xenograft invasion in mice was analyzed quantitatively (right), *P* value was calculated using the chi-square test. ***P < 0.001. **E** Representative immunohistochemical staining with anti-Ki67 in xenograft tumor sections (left, scale bars, 200 μm), the percentage of positive staining cells with the expression of Ki67 in xenografts were quantified (right). **F** Nude mice injected with silencing PTBP1 or silencing PTBP1 combined with overexpression of ITSN1-S LN229 cells. The images shown represent results for 8 mice. Red and blue dotted lines indicated the location and size of the tumors. **G** Tumor growth curve in indicated nude mice, values were expressed as mean ± SD (two-way ANOVA, ***P < 0.001). **H** The representative images show the xenograft tumors isolated from nude mice after sacrificed. **I** Representative images of hematoxylin-eosin staining in indicated tumor sections (left, scale bars, 200 μm), the frequency of xenograft invasion in mice was analyzed by quantitatively (right), *P* value was calculated using the chi-square test. ***P < 0.001. **J** Immunohistochemistry for Ki67 of representative images in tumor sections (left, scale bars, 200 μm), the expression of Ki67 was quantified with percentage of Ki67-positive cells to the total cells in tumor sections (right) (two-tailed Student’s *t* test, ***P < 0.001). **K** A proposed schema involving PTBP1 enhanced the inclusion of ITSN1 exon 30 to promote glioma progression. PTBP1 enhanced ITSN1 exon 30 inclusion through the binding of poly-pyrimidine sequences of exon 30, and the expression of ITSN1-S increased as well as the expression of ITSN1-L decreased, which promoted glioma progression.

In the following, we performed nude mice xenograft experiments. Consistently, ITSN1-L knockdown on top of PTBP1 silencing manifested no significant impact on tumor growth and final tumor volume compared with the control group (Fig. 7A–C). While silencing of ITSN1-L followed PTBP1 knockdown (shPTBP1/shITSN1-L/LN229) produced a statistically significant increase in the tumor invasion frequency compared to the control group (Fig. 7D). And the expression pattern of Ki67 was similar with the tumor volume in shPTBP1/shITSN1-L/LN229 mice group and control (Fig. 7E). Besides, ITSN1-S overexpression in PTBP1 knockdown LN229 cells (shPTBP1/ITSN1-S/LN229) restored the growth advantage (Fig. 7F–H) and the invasive ability compared to PTBP1 knockdown cells in mice (Fig. 7I). Similarly, ITSN1-S overexpression significantly dampened the inhibitory effect of shPTBP1 on the proliferation of glioma cells (Fig. 7J). Taken together, we concluded that PTBP1 promoted glioma progression by regulating alternative splicing of ITSN1. Finally, we summarized a proposed schema that PTBP1 enhanced the inclusion of ITSN1 exon 30 to promote glioma progression (Fig. 7K).

## Discussion

Dysregulated alternative splicing events are commonly observed in the development and progression of glioma. For example, alterations in alternative splicing of the *PKM*, *ANAX7*, and *MYO1B* genes have been implicated in the context of glioma progression [27–29]. Our work along with our previous studies provided solid evidence that two major isoforms of ITSN1 regulated by alternative splicing displayed opposite roles in glioma progression. And we found the ratio of ITSN1-S/ITSN1-L was positively correlated with glioma grading and poor prognosis. Our study demonstrated for the first time that PTBP1 activated the inclusion of ITSN1 exon 30 accompanied by upregulation of ITSN1-S/ITSN1-L ratio. Further, our studies revealed that PTBP1 promoted glioma progression by regulating alternative splicing of ITSN1, which further confirmed the important function of alternative splicing in cancer.

PTBP1 is a poly-pyrimidine tract-binding protein that plays an important role in alternative exon splicing and involved in a variety of cell processes [27]. Structurally, PTBP1 possesses four RNA Recognition Motif (RRM) domains, each of which binds the polypyrimidine sequence on the pre-mRNA [30]. Previous studies reported that the pyrimidine sequences recognized by each RBD in PTBP1 are slightly different [31, 32]. The recognized sequences by the RRM domain 1–4 are YCU, CU(N)N, YCUNN, YCN (Y indicating a pyrimidine and N any nucleotide) [32]. However, there is still no fully unified conclusion about the characteristic sequence of PTBP1 binding. Since PTBP1 binds to specific polypyrimidine sequences in pre-mRNA, it will affect alternative splicing of target genes. Therefore, determining the sequence of PTBP1 binding to the target gene could provide a theoretical basis for investigating the molecular mechanism of PTBP1 regulation of alternative splicing. In our study, the potential binding sequence of PTBP1 to *ITSN1* pre-mRNA was identified by analyzing CLIP-seq data. To further determine the interaction between PTBP1 and the potential binding site, we introduced the binding-site mutation and identified the binding-site mutation significantly suppressed PTBP1 binding affinity by RNA pull-down assay. Then, to examine whether the binding site was required for PTBP1 to promote the inclusion of ITSN1 exon 30, we constructed mutant minigene plasmid and elucidated that PTBP1 could efficiently regulate the alternative splicing of ITSN1.

PTBP1 commonly acts as a repressive splicing regulator [33]. Proposed mechanisms of repression include: (i) it binds to polypyrimidine sequences adjacent to variable exons to form loops in the corresponding regions of the pre-mRNA, leading to inhibiting the binding of splicing factors and the assembly of the splicing complex [32, 34, 35]; (ii) it specifically binds to the pyrimidine-rich sequence of the U1 snRNA stem-loop, inhibition of the ability of U1 snRNP to participate in the following assembly process of splicing complex A [36]; (iii) it binds to adjacent polypyrimidine sequences of variable exons and competitively inhibits U2AF2 binding, which in turn inhibits the assembly of splicing complex A [37]. But there was also a substantial number of PTBP1-activated splicing events [38], possibly by antagonizing other repressors [17, 39, 40]. In this study, our data confirmed that PTBP1 promoted the inclusion of ITSN1 exon 30, thus demonstrating that ITSN1 was one of the PTBP1-activated splicing targets. Therefore, the splicing regulation of ITSN1 may also depend on other splicing factors besides PTBP1. In addition, RNA splicing is exerted by a complex of spliceosomes involving five snRNPs (U1, U2, U4, U5, and U6). The detailed splicing regulation mechanism of ITSN1 mediated by PTBP1 requires further investigation.

Targeting alternative splicing in disease is gaining traction. Strategies to induce splice switching using splice-switching oligonucleotides (SSO) have yielded some progress in improving spinal muscular atrophy and muscular dystrophy both in mouse models and in clinical trials [41–43]. Unfortunately, SSO has poor intracellular uptake, which is a major obstacle to their usage as therapeutics [44]. Therefore, PTBP1, as the key splicing regulator of ITSN1, should be treated as a promising therapeutic target for suppressing the progress of malignant gliomas.

In summary, our present study demonstrated that the splicing factor PTBP1 promotes proliferation and mobility of glioma cells by controlling *ITSN1* pre-mRNA splicing and up-regulating the expression ratio of ITSN1-S/ITSN1-L. Modulation of ITSN1-S/ITSN1-L expression ratio by alternative splicing may highlight a new approach for the treatment of malignant gliomas.

## Supplementary information

Full and uncropped western blots images

Supplementary Figure legend

Supplementary Figure S1

Supplementary Figure S2

Supplementary Figure S3

Supplementary Figure S4

Supplementary Figure S5

Supplementary Figure S6

Supplementary Table S1

Supplementary Table S2

Supplementary Table S3

Supplementary Table S4

Supplementary Table S5

Supplementary Table S6

aj-checklist

## Data Availability

Some datasets presented in this study can be found in online repositories. The names of the repository/repositories and accession number(s) can be found in the article/supplementary material. Other datasets used and/or analyzed are available from the corresponding author on reasonable request.
