# Supplementary figures and images for: The alternative splicing of intersectin 1 regulated by PTBP1 promotes human glioma progression
Source: Cell Death Dis. 2022 Sep 28;13(9):835. doi: 10.1038/s41419-022-05238-1 (PMC9519902; doi:10.1038/s41419-022-05238-1)

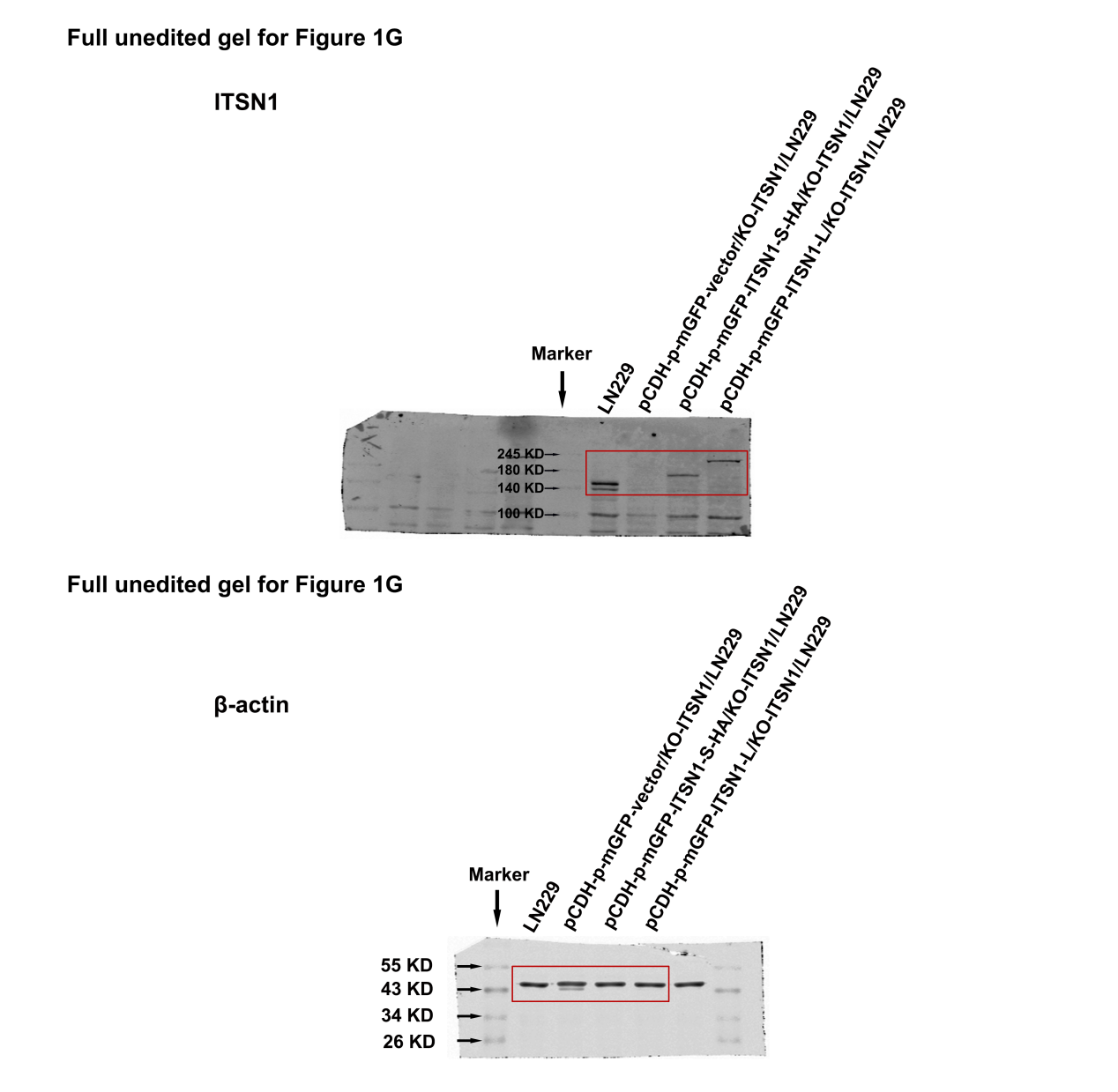


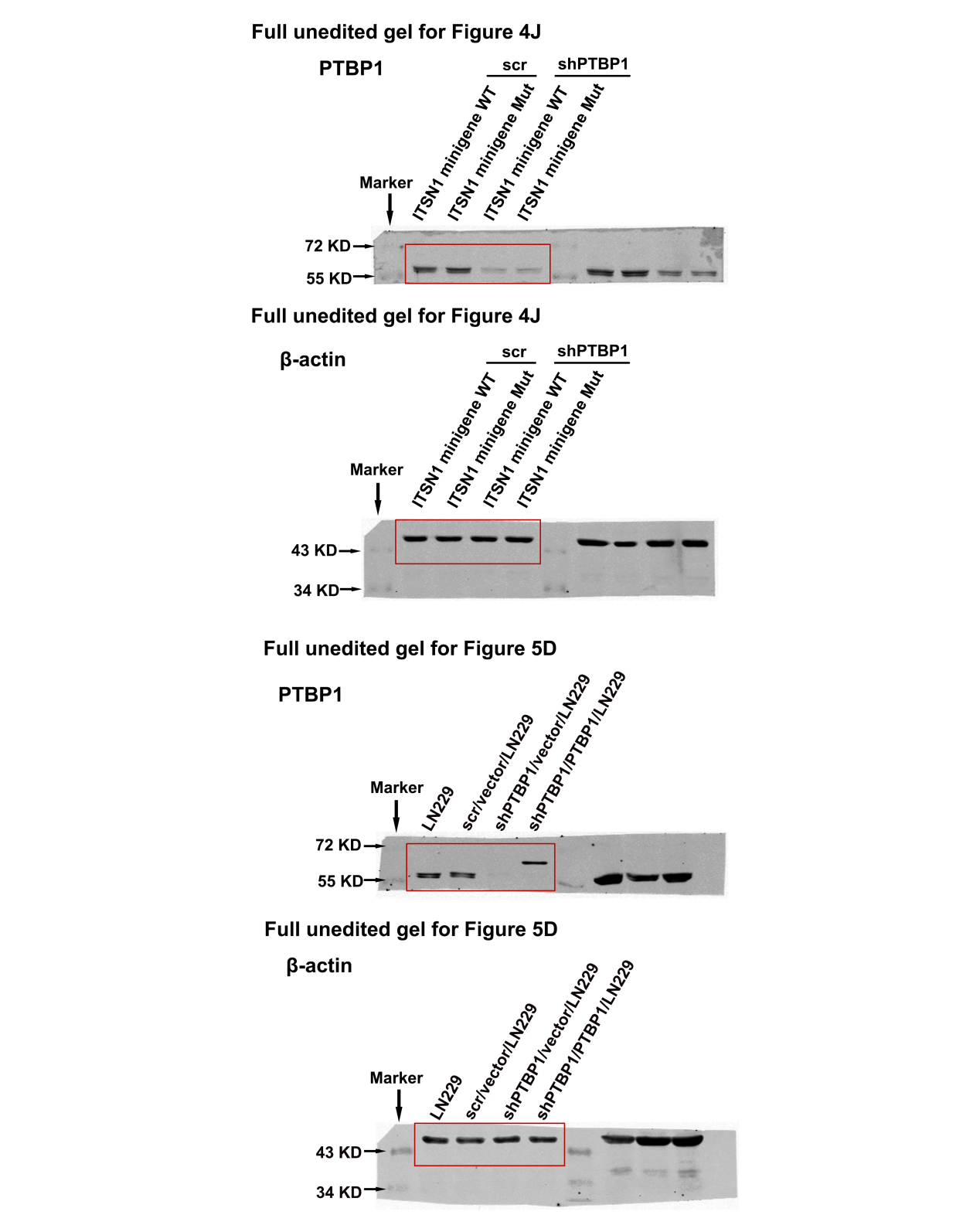

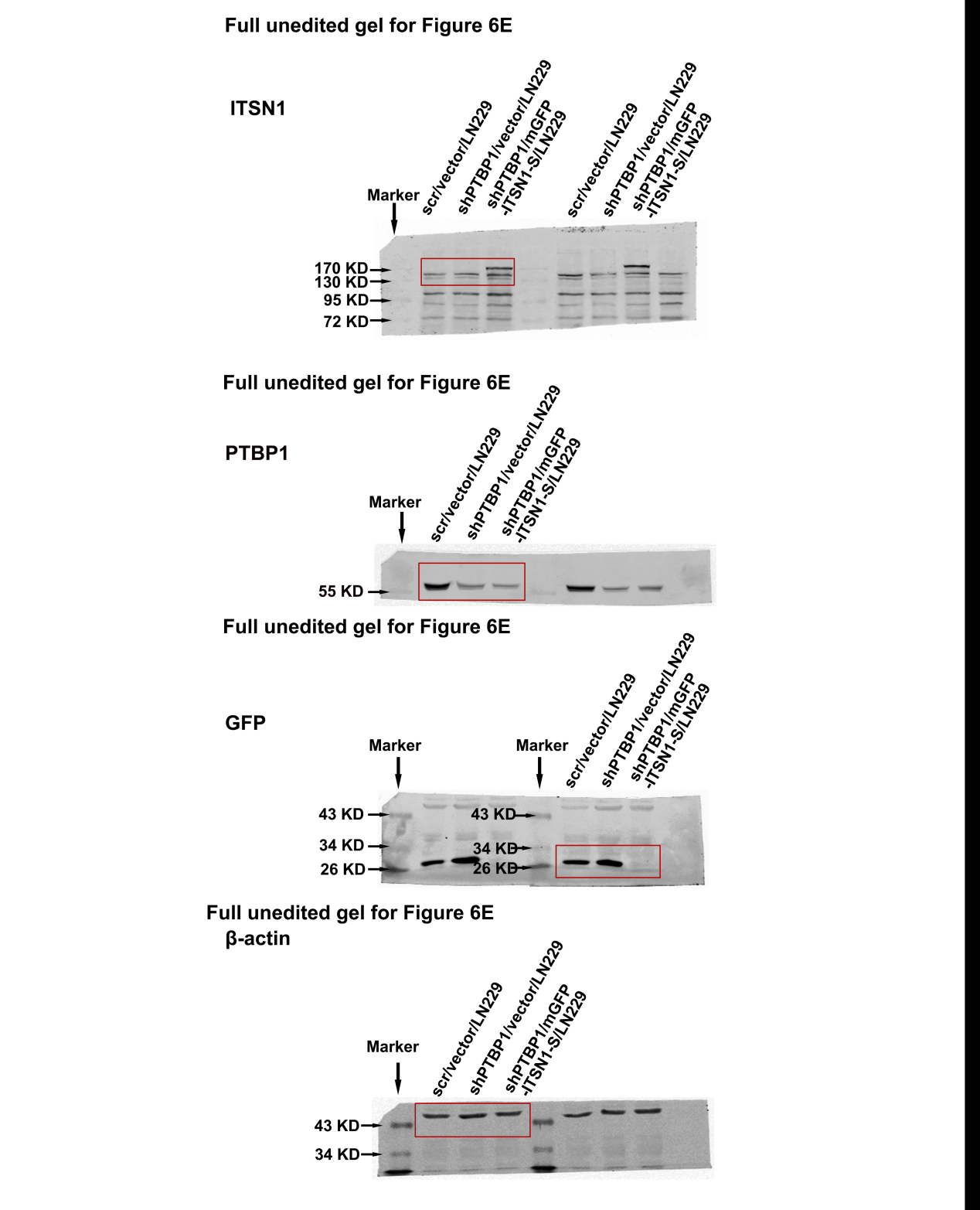

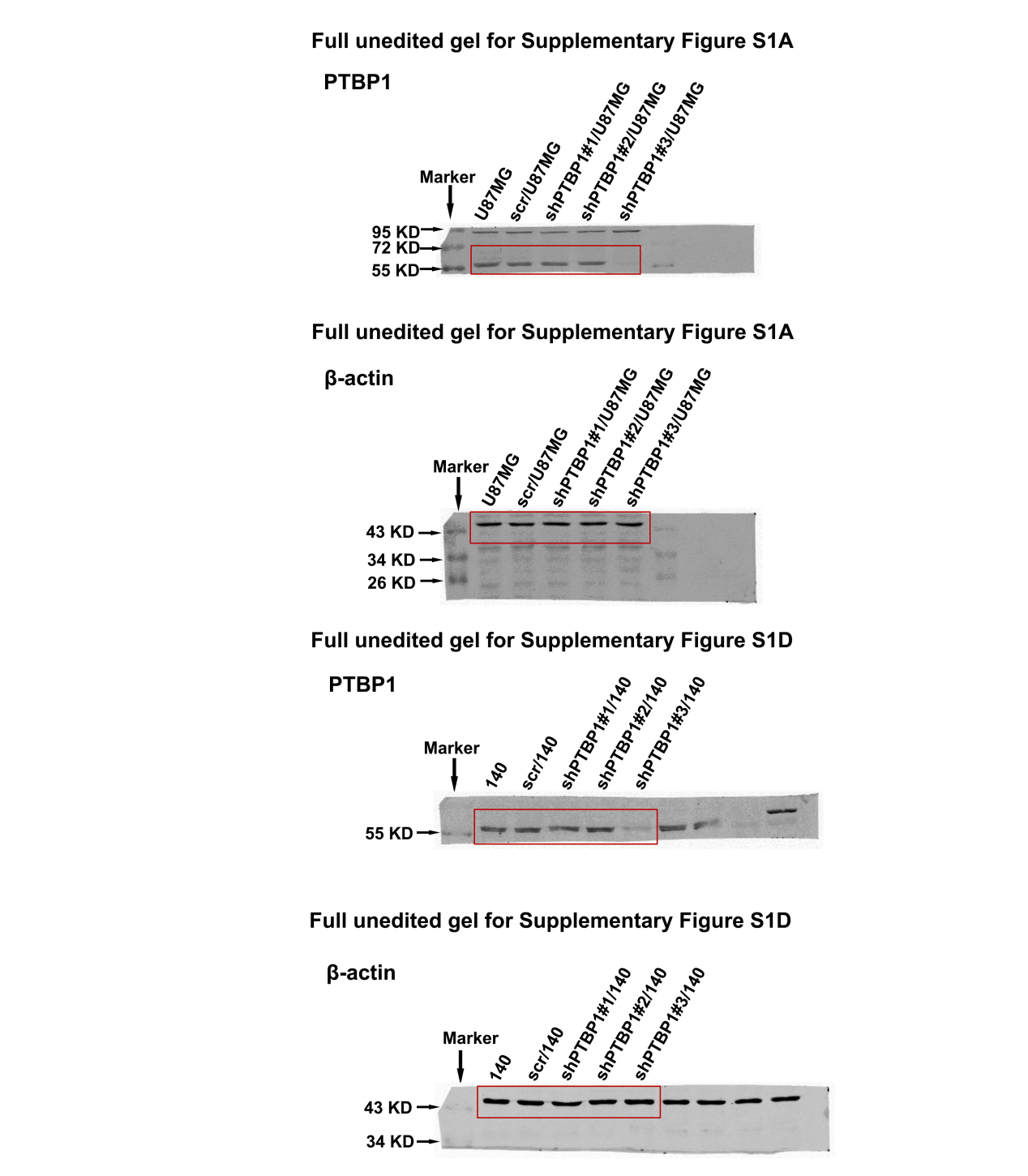

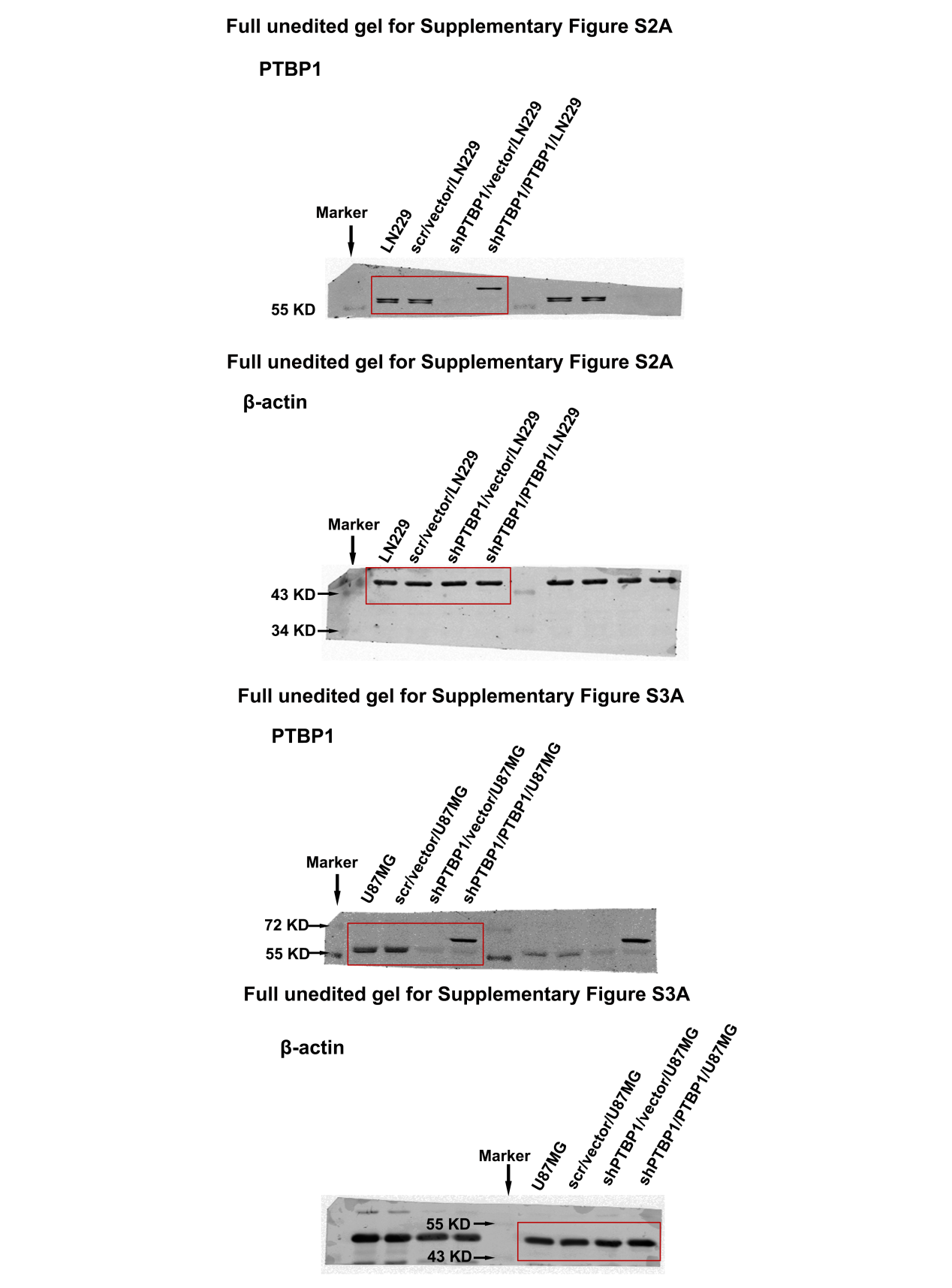

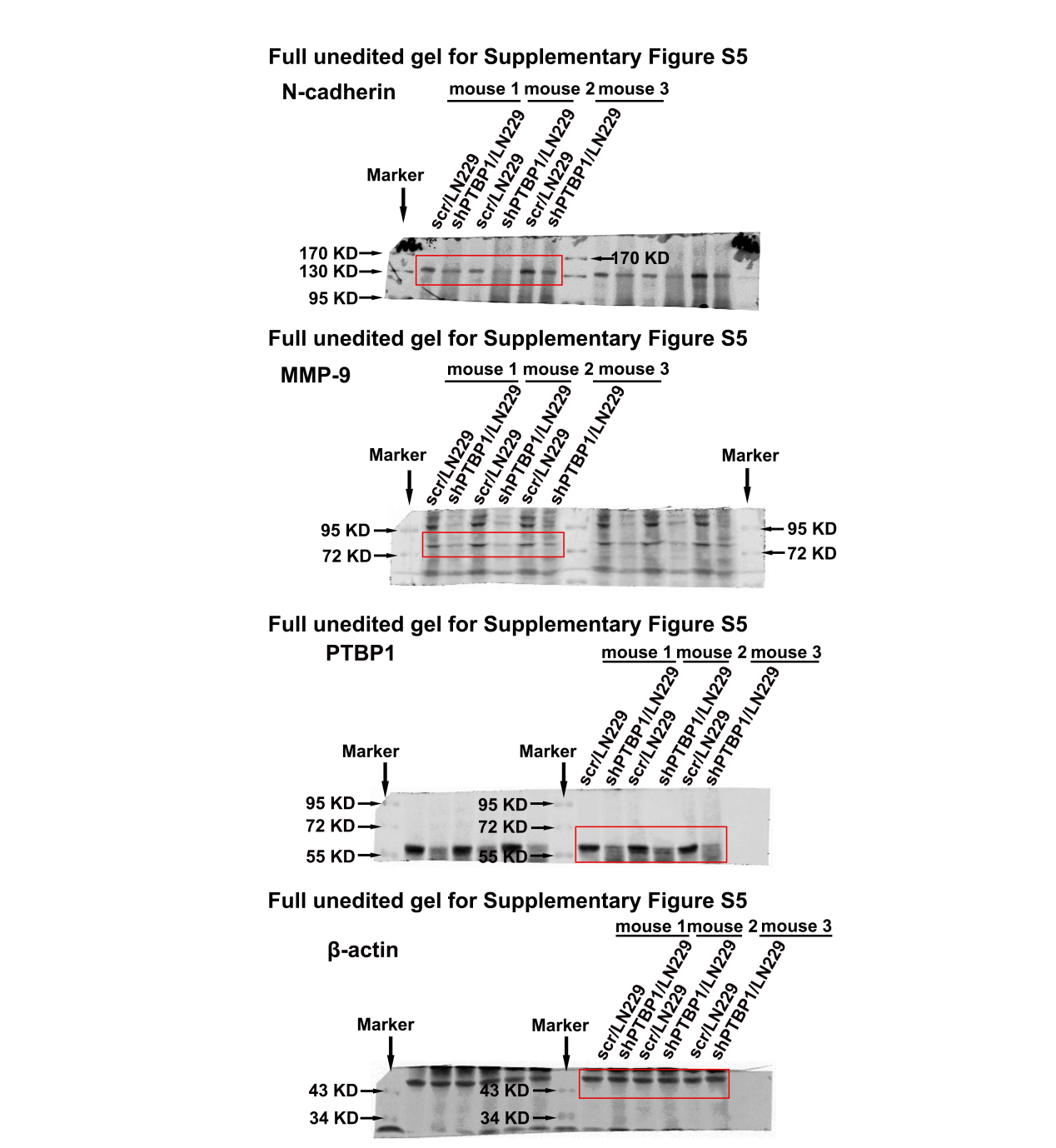

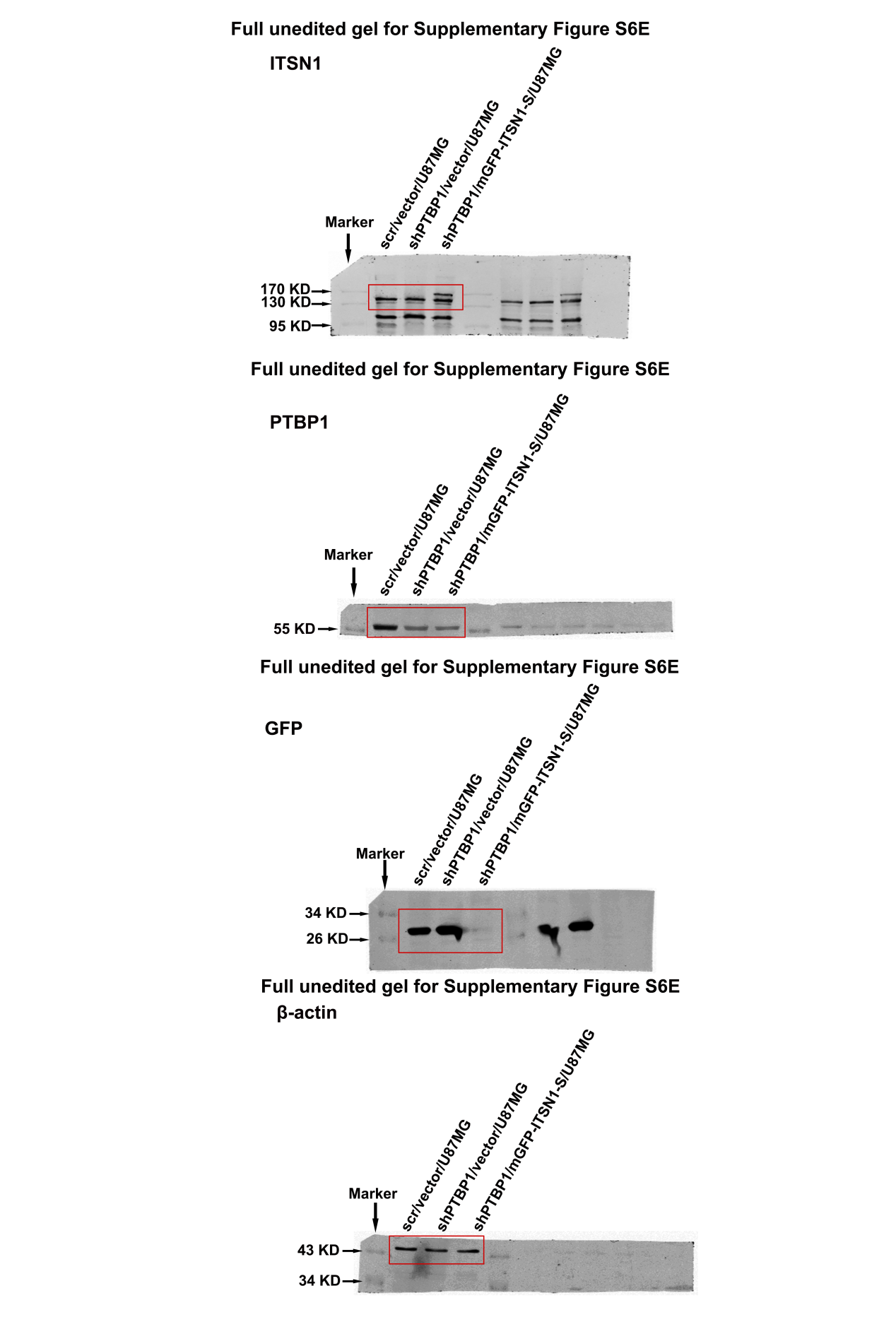

Supplement: Supplementary file 1 — Full and uncropped western blots images [file 41419_2022_5238_MOESM1_ESM.docx]

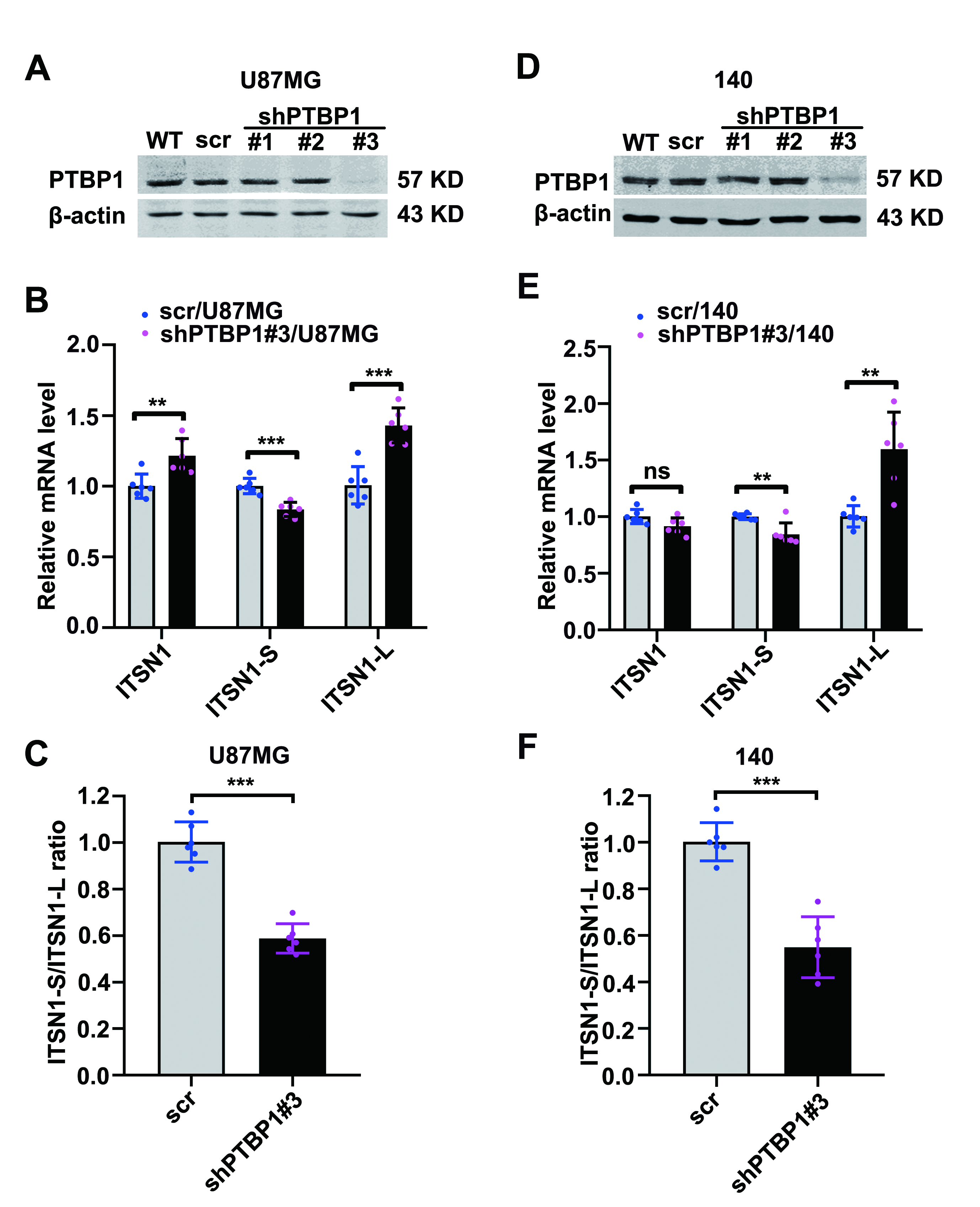

Supplement: Supplementary file 3 — Supplementary Figure S1 [file 41419_2022_5238_MOESM3_ESM.tif]

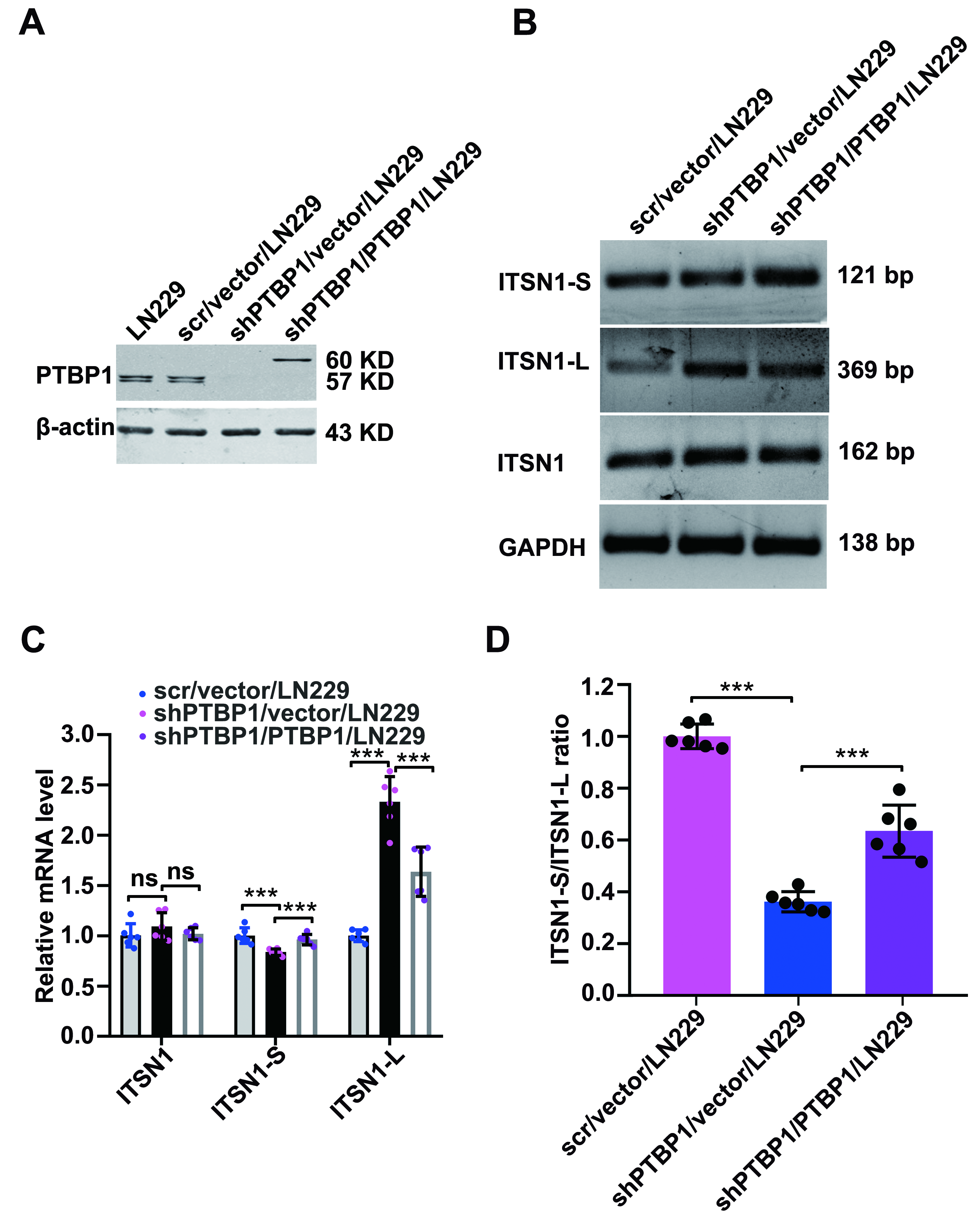

Supplement: Supplementary file 4 — Supplementary Figure S2 [file 41419_2022_5238_MOESM4_ESM.tif]

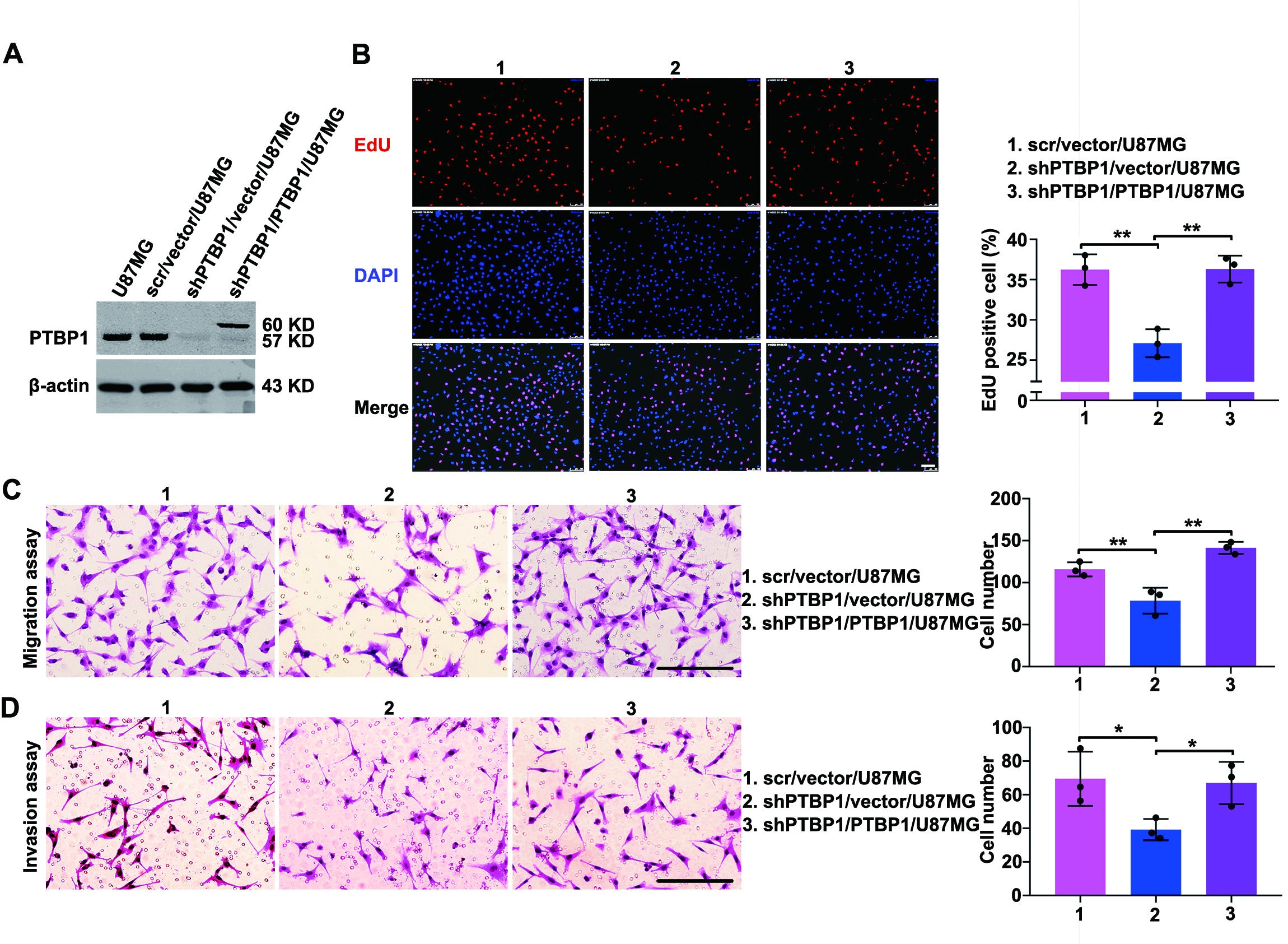

Supplement: Supplementary file 5 — Supplementary Figure S3 [file 41419_2022_5238_MOESM5_ESM.tif]

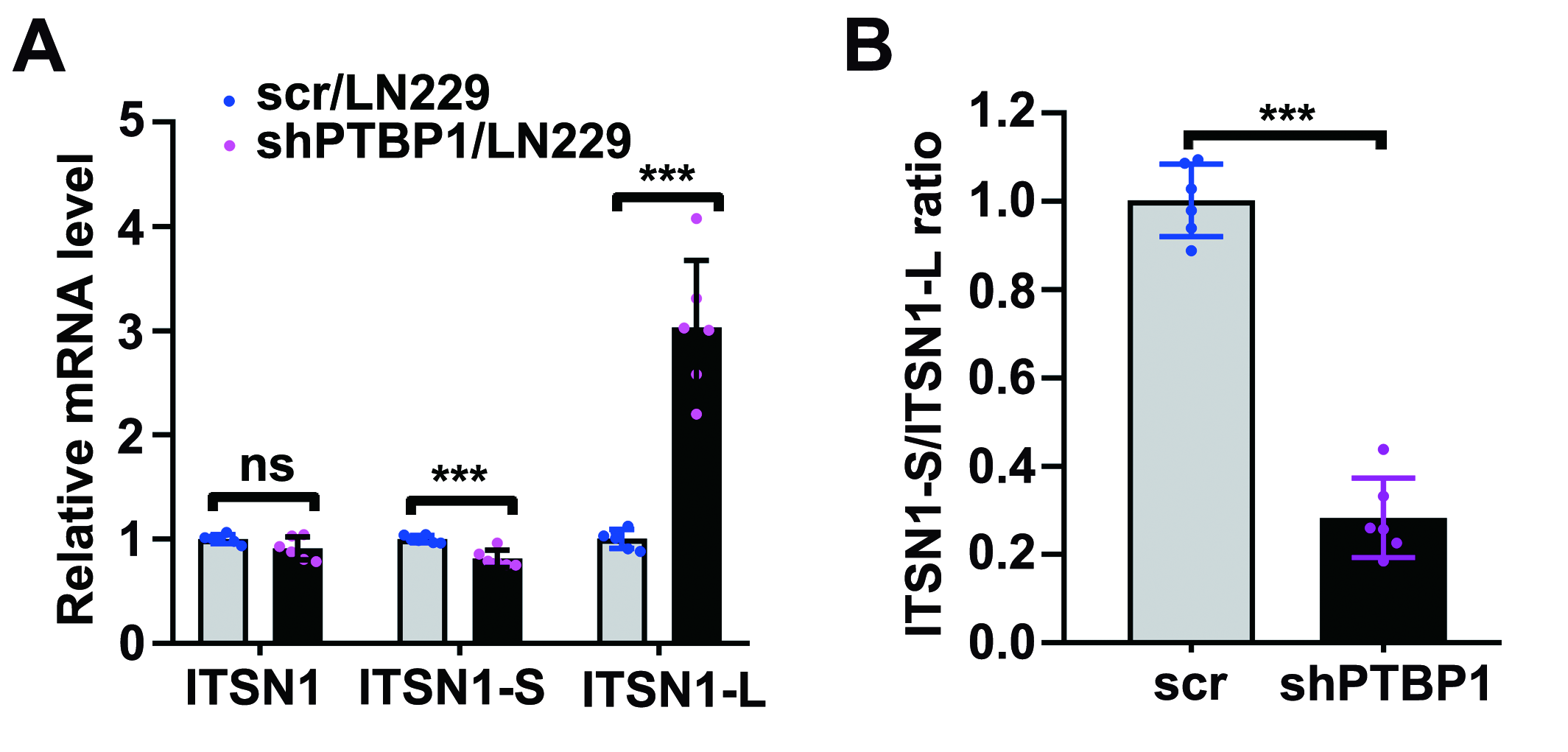

Supplement: Supplementary file 6 — Supplementary Figure S4 [file 41419_2022_5238_MOESM6_ESM.tif]

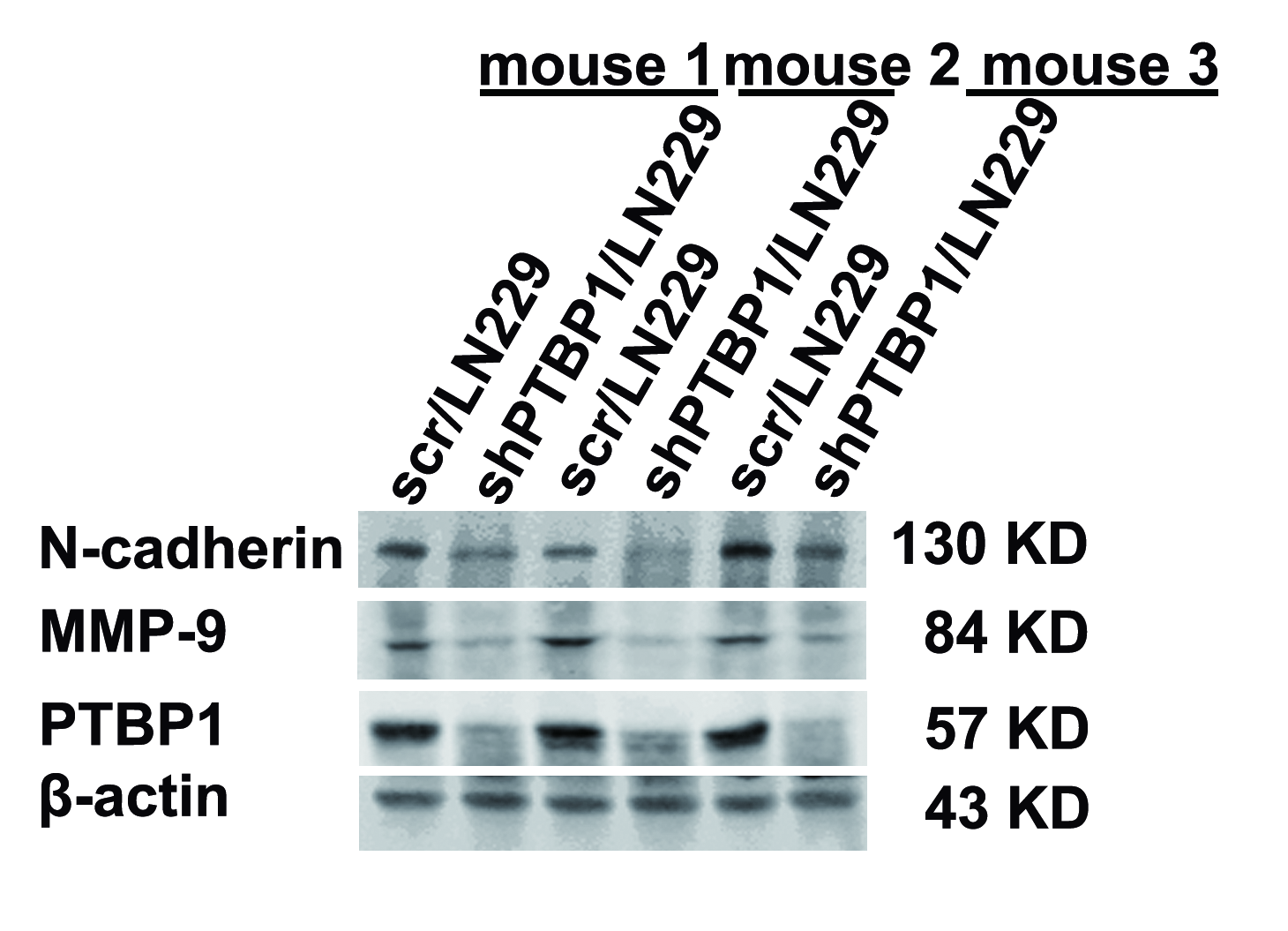

Supplement: Supplementary file 7 — Supplementary Figure S5 [file 41419_2022_5238_MOESM7_ESM.tif]

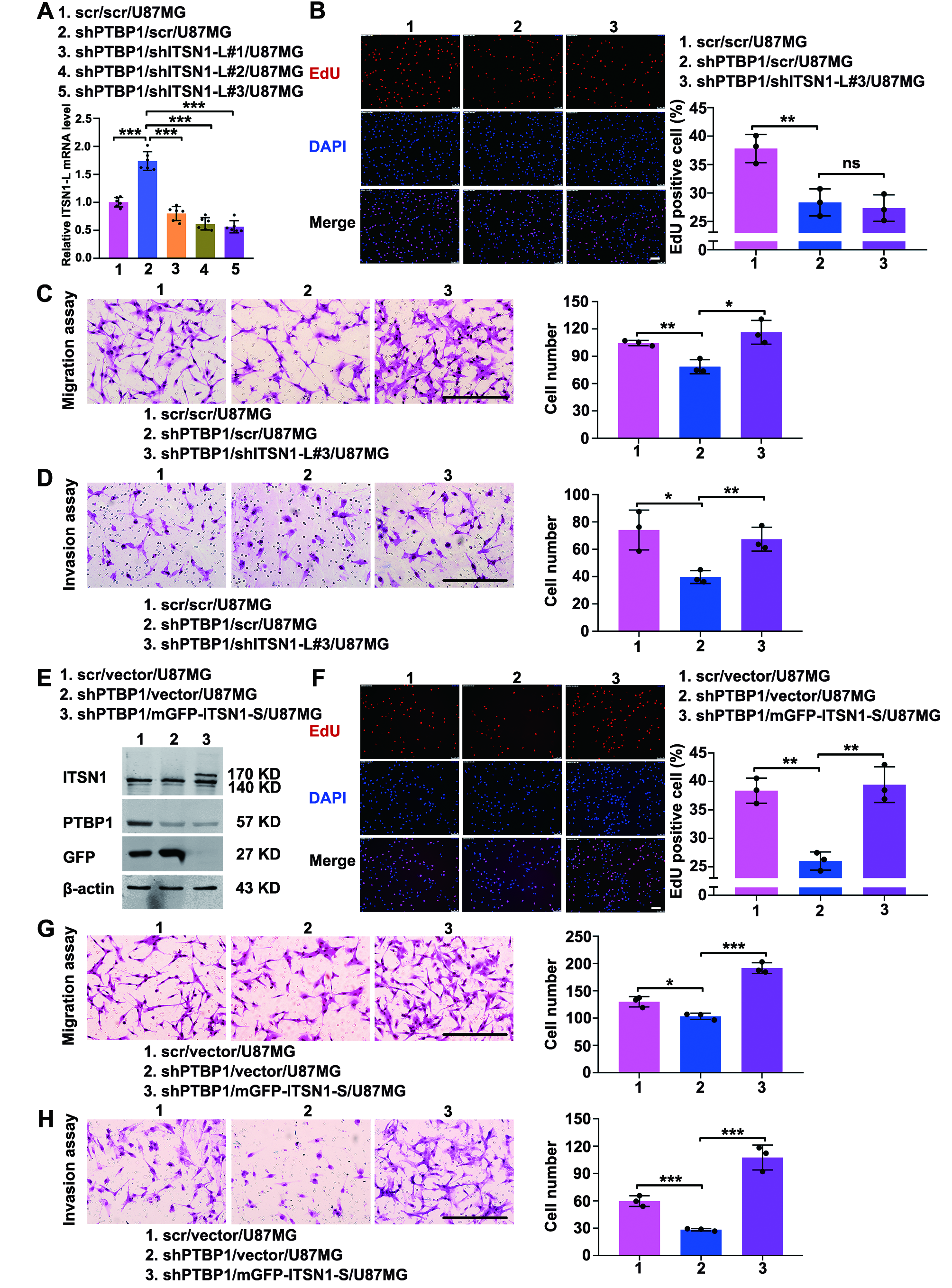

Supplement: Supplementary file 8 — Supplementary Figure S6 [file 41419_2022_5238_MOESM8_ESM.tif]
