# Supplementary material for: The alternative splicing of intersectin 1 regulated by PTBP1 promotes human glioma progression
Source: Cell Death Dis. 2022 Sep 28;13(9):835. doi: 10.1038/s41419-022-05238-1 (PMC9519902; doi:10.1038/s41419-022-05238-1)
Supplement: Supplementary file 2 — Supplementary Figure legend [file 41419_2022_5238_MOESM2_ESM.docx]

**Supplementary Figure legend**

**Supplementary Figure S1**

**Knockdown of PTBP1 down-regulated the ratio of ITSN1-S/ITSN1-L.** (**A**) Establishment and verification of stable cell lines underexpressing PTBP1 in U87MG cells. β-actin was used as an internal control. (**B**) RT-qPCR analysis showing changes in the ITSN1-S, ITSN1-L and total ITSN1 mRNA level upon silencing PTBP1 in U87MG cells. (**C**) RT-qPCR analysis showing changes in the ratio of ITSN1-S/ITSN1-L upon silencing PTBP1 in U87MG cells. (**D**) Establishment and verification of stable cell lines underexpressing PTBP1 in 140 cells. β-actin was used as an internal control. (**E**) RT-qPCR analysis showing changes in the ITSN1-S, ITSN1-L and total ITSN1 mRNA level upon silencing PTBP1 in 140 cells. (**F**) RT-qPCR analysis showing changes in the ratio of ITSN1-S/ITSN1-L upon silencing PTBP1 in 140 cells. Values were expressed as mean ± SD from six independent experiments in Figure S1 **B**-**C** and **E**-**F** (two-tailed Student’s *t* test, ***P* < 0.01, ****P* < 0.001, ns, no significance).

**Supplementary Figure S2**

**Silencing PTBP1 down-regulated the ratio of ITSN1-S/ITSN1-L.** (**A**) Western blot analysis of PTBP1 knockdown and rescue in the extracts of cells as indicated. (**B**) Representative pictures of agarose PCR gels depicting the inversion in the splicing pattern of ITSN1 regulated by PTBP1 after PTBP1 silence and rescue in LN229 cell line. (**C**) RT-qPCR analysis showing changes in the ITSN1-S, ITSN1-L and total ITSN1 mRNA level after PTBP1 knockdown and rescue in LN229 cell line. (**D**) RT-qPCR analysis showing changes in the ratio of ITSN1-S/ITSN1-L after PTBP1 knockdown and rescue in LN229 cell line. Values were expressed as mean ± SD from six independent experiments in Figure S2 **C** and **D** (two-tailed Student’s *t* test, ****P* < 0.001, ns, no significance).

**Supplementary Figure S3**

**PTBP1 promoted proliferation, migration and invasion of glioma cells *in vitro*.** (**A**) Western blot analysis of PTBP1 knockdown and rescue in the extracts of U87MG cells. Loading control: β-actin. (**B**) Images of EdU staining (left, scale bars, 100 μm) and the comparison of EdU-positive rates between the control and silencing PTBP1 cells (right). (**C**) Migration assay of the indicated cells. Cells migrating through transwell inserts were stained, photographed, and quantified. (**D**) Invasion assay results. Cells invading through matrigel matrix-coated transwell inserts were stained, photographed, and quantified. Values were expressed as mean ± SD from three independent experiments in Figure S3 **B-D** (two-tailed Student’s t test, **P* < 0.05, ***P* <0.01).

**Supplementary Figure S4**

**Silencing PTBP1 down-regulated the ratio of ITSN1-S/ITSN1-L *in vivo*.** (**A**) RT-qPCR analysis showing changes in the ITSN1-S, ITSN1-L and total ITSN1 mRNA level after PTBP1 knockdown in xenograft tumor tissues. (**B**) RT-qPCR analysis showing changes in the ratio of ITSN1-S/ITSN1-L after PTBP1 knockdown in xenograft tumor tissues.

**Supplementary Figure S5**

**Silencing PTBP1 impaired the invasion of glioma cells *in vivo*.** Western blotting analysis of PTBP1, N-cadherin, and MMP-9 expression in 3 mice tumor tissues formed by scr/LN229 cells and shPTBP1/LN229 cells. β-actin was used as a loading control.

**Supplementary Figure S6**

**PTBP1 promoted proliferation, migration and invasion of glioma cells by regulating alternative splicing of ITSN1 *in vitro*.** (**A**) Establishment and verification of stable cell lines silencing PTBP1 followed by knocking down of ITNS1-L in U87MG cells. Values were expressed as mean ± SD from six independent experiments (two-tailed Student’s *t* test, ****P* <0.001). (**B**) Images of EdU staining (left, scale bars, 100 μm) and the comparison of EdU-positive rates among indicated cells (right). (**C**) Migration assay of the indicated cells. Cells migrating through transwell inserts were stained, photographed, and quantified. Scale bars, 200 μm. (**D**) Invasion assay results. Cells invading through matrigel matrix-coated transwell inserts were stained, photographed, and quantified. Scale bars, 200 μm. (**E**) Western blot analysis of stable cell lines silencing PTBP1 followed by over-expression of ITSN1-S in U87MG. (**F**) Images of EdU staining (left, scale bars, 100 μm) and the comparison of EdU-positive rates among indicated cells (right). (**G**) Migration assay of the indicated cells. Cells migrating through transwell inserts were stained, photographed, and quantified. Scale bars, 200 μm. (**H**) Invasion assay of the indicated cells. Cells invading through matrigel matrix-coated transwell inserts were stained, photographed, and quantified. Scale bars, 200 μm. Values were expressed as mean ± SD from three independent experiments in Figure S5 **B-D** and **F-H** (two-tailed Student’s *t* test, **P* < 0.05, ***P* <0.01, ****P* <0.001, ns, no significance).
